# Mechanism of Neuroprotective Mitochondrial Remodeling by PKA/AKAP1

**DOI:** 10.1371/journal.pbio.1000612

**Published:** 2011-04-19

**Authors:** Ronald A. Merrill, Ruben K. Dagda, Audrey S. Dickey, J. Thomas Cribbs, Steven H. Green, Yuriy M. Usachev, Stefan Strack

**Affiliations:** 1 Department of Pharmacology, University of Iowa, Iowa City, Iowa, United States of America; 2 Department of Biological Sciences, University of Iowa, Iowa City, Iowa, United States of America; St. Jude Children's Research Hospital, United States of America

## Abstract

Mitochondrial shape is determined by fission and fusion reactions catalyzed by large GTPases of the dynamin family, mutation of which can cause neurological dysfunction. While fission-inducing protein phosphatases have been identified, the identity of opposing kinase signaling complexes has remained elusive. We report here that in both neurons and non-neuronal cells, cAMP elevation and expression of an outer-mitochondrial membrane (OMM) targeted form of the protein kinase A (PKA) catalytic subunit reshapes mitochondria into an interconnected network. Conversely, OMM-targeting of the PKA inhibitor PKI promotes mitochondrial fragmentation upstream of neuronal death. RNAi and overexpression approaches identify mitochondria-localized A kinase anchoring protein 1 (AKAP1) as a neuroprotective and mitochondria-stabilizing factor in vitro and in vivo. According to epistasis studies with phosphorylation site-mutant dynamin-related protein 1 (Drp1), inhibition of the mitochondrial fission enzyme through a conserved PKA site is the principal mechanism by which cAMP and PKA/AKAP1 promote both mitochondrial elongation and neuronal survival. Phenocopied by a mutation that slows GTP hydrolysis, Drp1 phosphorylation inhibits the disassembly step of its catalytic cycle, accumulating large, slowly recycling Drp1 oligomers at the OMM. Unopposed fusion then promotes formation of a mitochondrial reticulum, which protects neurons from diverse insults.

## Abstract 2

The mitochondrial signaling complex PKA/AKAP1 protects neurons against mitochondrial fragmentation and cell death by phosphorylating and inactivating the mitochondrial fission enzyme Drp1.

## Author Summary

Mitochondria, the cellular powerhouse, are highly dynamic organelles shaped by opposing fission and fusion events. Research over the past decade has identified many components of the mitochondrial fission/fusion machinery and led to the discovery that mutations in genes coding for these proteins can cause human neurological diseases. While it is well established that mitochondrial shape changes are intimately involved in cellular responses to environmental stressors, we know very little about the mechanisms by which cells dynamically adjust mitochondrial form and function. In this report, we show that the scaffold protein AKAP1 brings the cAMP-dependent protein kinase PKA to the outer mitochondrial membrane to protect neurons from injury. The PKA/AKAP1 complex functions by inhibiting Drp1, an enzyme that mechanically constricts and eventually severs mitochondria. Whereas active, dephosphorylated Drp1 rapidly cycles between cytosol and mitochondria, phosphorylated Drp1 builds up in inactive mitochondrial complexes, allowing mitochondria to fuse into a neuroprotective reticulum. Our results suggest that altering the balance of kinase and phosphatase activities at the outer mitochondrial membrane may provide the basis for novel neuroprotective therapies.

## Introduction

Opposing fission and fusion events determine the shape and interconnectivity of mitochondria to regulate various aspects of their function, including ATP production, Ca^++^ buffering, free radical homeostasis, mitochondrial DNA inheritance, and organelle quality control. In addition, fragmentation of neuronal mitochondria is necessary for their transport to and proper development and function of synapses. Moreover, mitochondrial fission is an early step in the mitochondrial apoptosis pathway, and inhibiting fission can block or delay apoptosis in a variety of cell types, including neurons [1]–[3].

Fission and fusion processes are catalyzed by large GTPases of the dynamin superfamily. Mitochondrial fission requires dynamin-related protein 1 (Drp1), which, similar to the “pinchase” dynamin, is thought to mechanically constrict and eventually sever mitochondria. Normally a largely cytosolic protein, Drp1 is recruited to the outer mitochondrial membrane (OMM) by a poorly characterized multiprotein complex that includes the transmembrane proteins Fis1 and Mff [4]–[6]. Mitochondrial fusion is carried out by the concerted action of OMM-anchored GTPases (mitofusin-1 and -2 in vertebrates) and optic atrophy 1 (Opa1), a GTPase localized to the intermembrane space [4]. A properly controlled fission/fusion balance appears to be particularly critical in neurons, since mutations in mitochondrial fission/fusion enzymes are responsible for common neurological disorders in humans [7]–[10]. All mitochondria-restructuring enzymes are essential for mammalian development, as mice that lack Drp1, Opa1, or either of the two mitofusins die during early embryogenesis [11]–[14].

Our understanding of the signaling events that regulate this group of organelle shaping GTPases is limited. Drp1, in particular, is subject to complex posttranslational modification by ubiquitylation, sumoylation, nitrosylation, and phosphorylation [15],[16]. Highly conserved among metazoan Drp1 orthologs, the two characterized serine phosphorylation sites are located 20 amino acids apart, bordering the C-terminal GTPase effector domain (GED). Since the numbering differs between Drp1 orthologs and splice variants, we will refer to these sites by the kinase first shown to target them, rather than their sequence number. Ser_CDK_ (Ser616 in human, Ser635 in rat splice variant 1) is phosphorylated by the cyclin-dependant kinase 1/cyclin B complex, leading to fragmentation of the mitochondrial network during mitosis. Phosphorylation of Ser_PKA_ (Ser637 in human, Ser656 in rat splice variant 1) is mediated by both PKA and Ca^2+^/calmodulin-dependent protein kinase I (CaMKI). Three laboratories, including ours, found that Ser_PKA_ phosphorylation by PKA promotes mitochondrial elongation presumably through Drp1 inhibition [17]–[19], whereas a fourth group reported the opposite effect upon targeting of the same site by CaMKI [20].

We previously showed that Bβ2, a neuron-specific and OMM-targeted regulatory subunit of protein phosphatase 2A (PP2A), sensitizes neurons to various insults by promoting mitochondrial fission [21]. Here, we identify outer mitochondrial PKA as the opposing fusion and survival promoting kinase. Evidence for a model is presented in which the multifunctional scaffold protein AKAP1 recruits PKA to the OMM to phosphorylate Drp1 at Ser_PKA_. Phosphorylation traps Drp1 in large, slowly recycling complexes, allowing mitochondria to fuse into a neuroprotective reticulum.

## Results

### cAMP and Outer Mitochondrial PKA Activity Promote Mitochondrial Elongation

**Figure 1:**
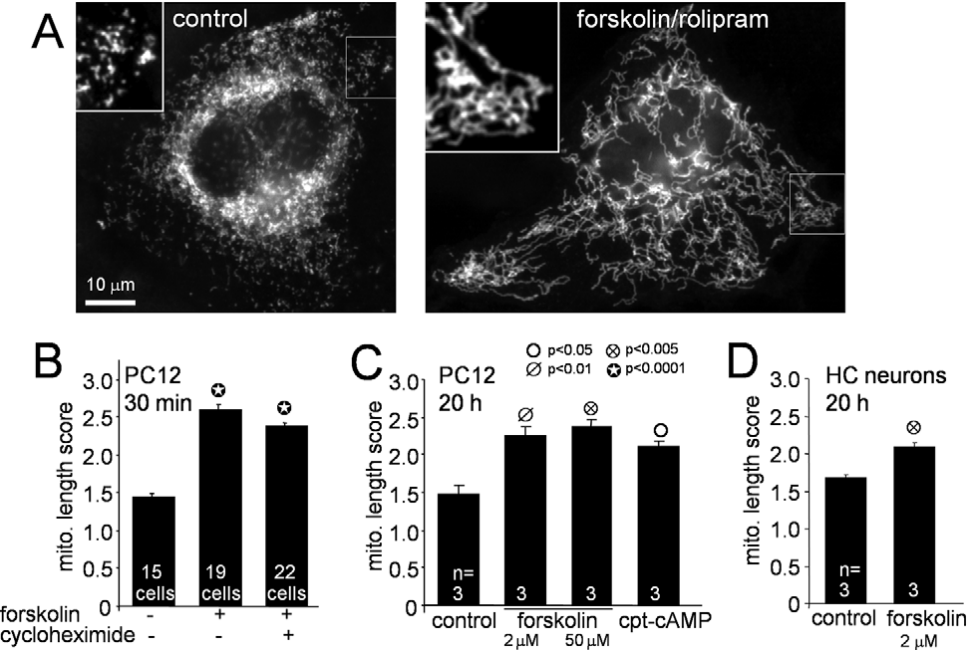
PKA activators rapidly induce mitochondrial fusion. TMRM or MitoTracker-stained PC12 cells (A–C) or hippocampal neurons (D) were treated for the indicated times with either vehicle or the listed compounds and mitochondrial morphology was quantified by blinded comparison to reference images (B–D, means ± S.E.M.). (A) Representative epifluorescence images show formation of interconnected mitochondria upon treatment of PC12 cells with forskolin/rolipram (forsk/roli, 20 µM/1 µM, 3 h). In the representative experiment shown in (B), rapid mitochondrial elongation by 50 µM forskolin is not affected by 100 µg/ml cycloheximide to inhibit protein synthesis. Long-term (20 h) forskolin or a cell-permeant cAMP analog (200 µM cpt-cAMP) promotes mitochondrial fusion in PC12 cells (C) and hippocampal neurons (D); summaries of three independent experiments with 20–30 cells per condition are shown.

In an effort to identify signal transduction pathways that alter the mitochondrial fission/fusion balance, we investigated the effect of elevating cAMP levels in PC12 pheochromocytoma cells and primary hippocampal neurons. Mitochondria were visualized by transfection with mitochondria-targeted GFP or by live-staining with the fluorescent dye MitoTracker Red. Application of the adenylate cyclase agonist forskolin either alone or in combination with the phosphodiesterase type IV inhibitor rolipram resulted in the conversion of mostly punctiform or short and stubby mitochondria to highly elongated and interconnected organelles (Figure 1A–D). Mitochondrial shape changes were quantified by image analysis [22] as well as by blinded comparison to a set of reference images (length score 0 to 4; Figure S1A; [21]). Both methods yielded comparable elongation measures that correlated on a cell-by-cell basis (Figure S1B, C, and D). Mitochondrial elongation was readily apparent within 30 min of forskolin addition and was insensitive to inhibition of protein synthesis by cycloheximide, which together suggests non-genomic actions of cAMP (Figure 1B). Mitochondrial elongation mediated by forskolin or the membrane permeable cAMP analog cpt-cAMP lasted for at least 20 h in PC12 cells and in the soma of hippocampal neurons (Figure 1C,D).

**Figure 2:**
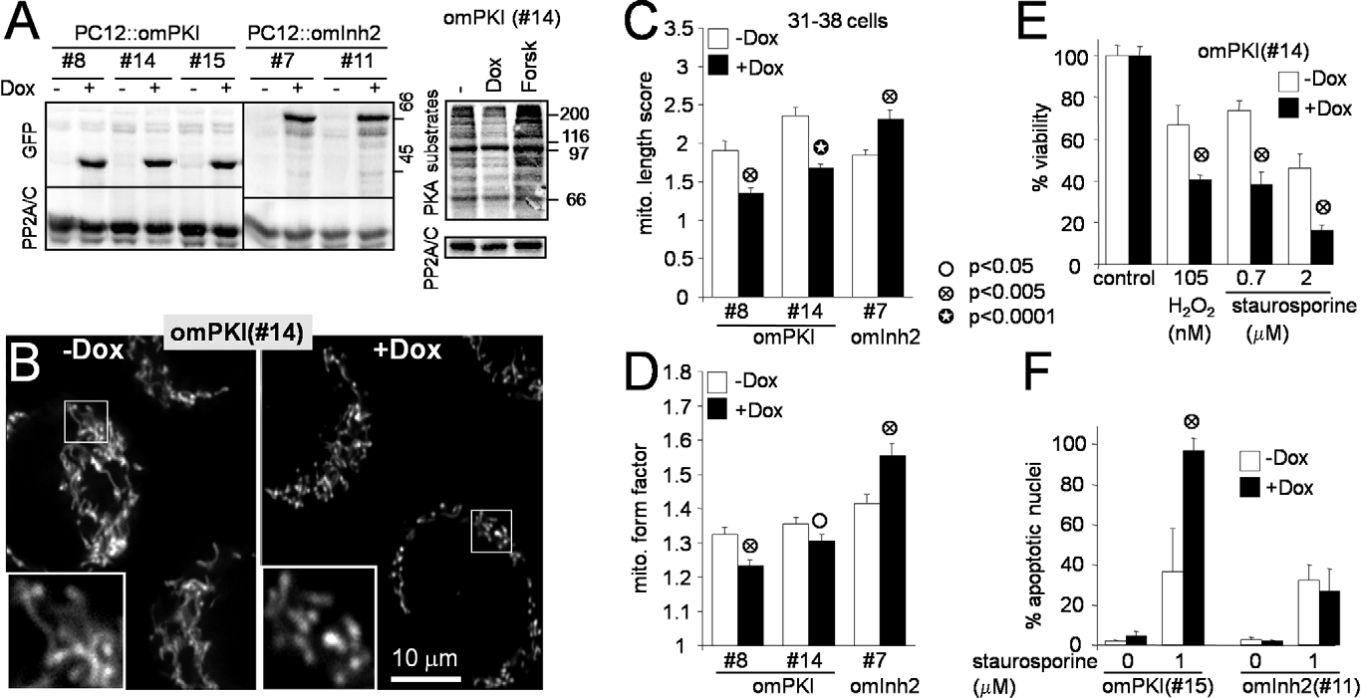
Inducible inhibition of outer mitochondrial PKA antagonizes mitochondrial fusion and survival. (A) Lysates of clonal PC12 cell lines (clone numbers listed) expressing outer-mitochondrial GFP-PKI (omPKI) or GFP-inhibitor-2 (omInh2) from a doxycycline (Dox)-inducible promoter were treated ± Dox (1 µg/ml, 48 h) or forskolin (forsk, 10 µM, 2 h) and immunoblotted for GFP, PKA substrates (RXX[pS/pT] antibody), and PP2A/C as a loading control. (B–D) PC12::omPKI and omInh2 cells were analyzed for mitochondrial morphology ± Dox induction for 2 d (representative confocal images of live cells stained with TMRM (B), reference image based length scores in (C), and digital morphometry in (D) of the same set of 31–38 cells). (E, F) PC12 cell lines were treated ± Dox for 2 d to induce omPKI or omInh2, followed by a 2 d challenge with staurosporine or H_2_O_2_. In (E), viability was scored by a colorimetric assay (tetrazolium reduction to formazan), while apoptotic nuclei were counted in (F). Bar graphs show means ± S.E.M. and are representative of at least three independent experiments. Student's *t* test comparisons are between ± Dox-treated cultures.

To implicate PKA in the morphogenetic effects of cAMP, we established clonal PC12 cell lines that inducibly express the PKA inhibitory peptide PKI fused to GFP and the outer mitochondrial anchor domain of MAS70p (omPKI). Cells with inducible expression of an OMM-directed form of the protein phosphatase 1 (PP1) modulator inhibitor-2 (omInh2) were analyzed for comparison (Figure 2A). Induction of omPKI by doxycycline resulted in mitochondrial fragmentation, measured both by subjective scoring and image analysis. Conversely, mitochondria decorated with the PP1 inhibitor were significantly elongated compared to mitochondria of uninduced cells (Figure 2B–D).

Given that mitochondrial fragmentation is associated with apoptotic and non-apoptotic cell death [2],[3], we examined cell lines expressing mitochondria-targeted PP1 and PKA inhibitors for their susceptibility to apoptotic stressors. Inhibition of neither outer mitochondrial PP1 nor PKA affected survival or growth of PC12 cells under basal conditions (Figure 2F and unpublished data). In contrast, induction of omPKI was associated with increased sensitivity to several apoptosis inducers, including H_2_O_2_ and staurosporine (Figure 2E,F). These results indicate that PKA activity at the mitochondrial surface opposes fragmentation of this organelle and increases resistance to apoptosis inducers.

### PKA/AKAP1 Supports Neuronal Survival and Mitochondrial Network Integrity

**Figure 3:**
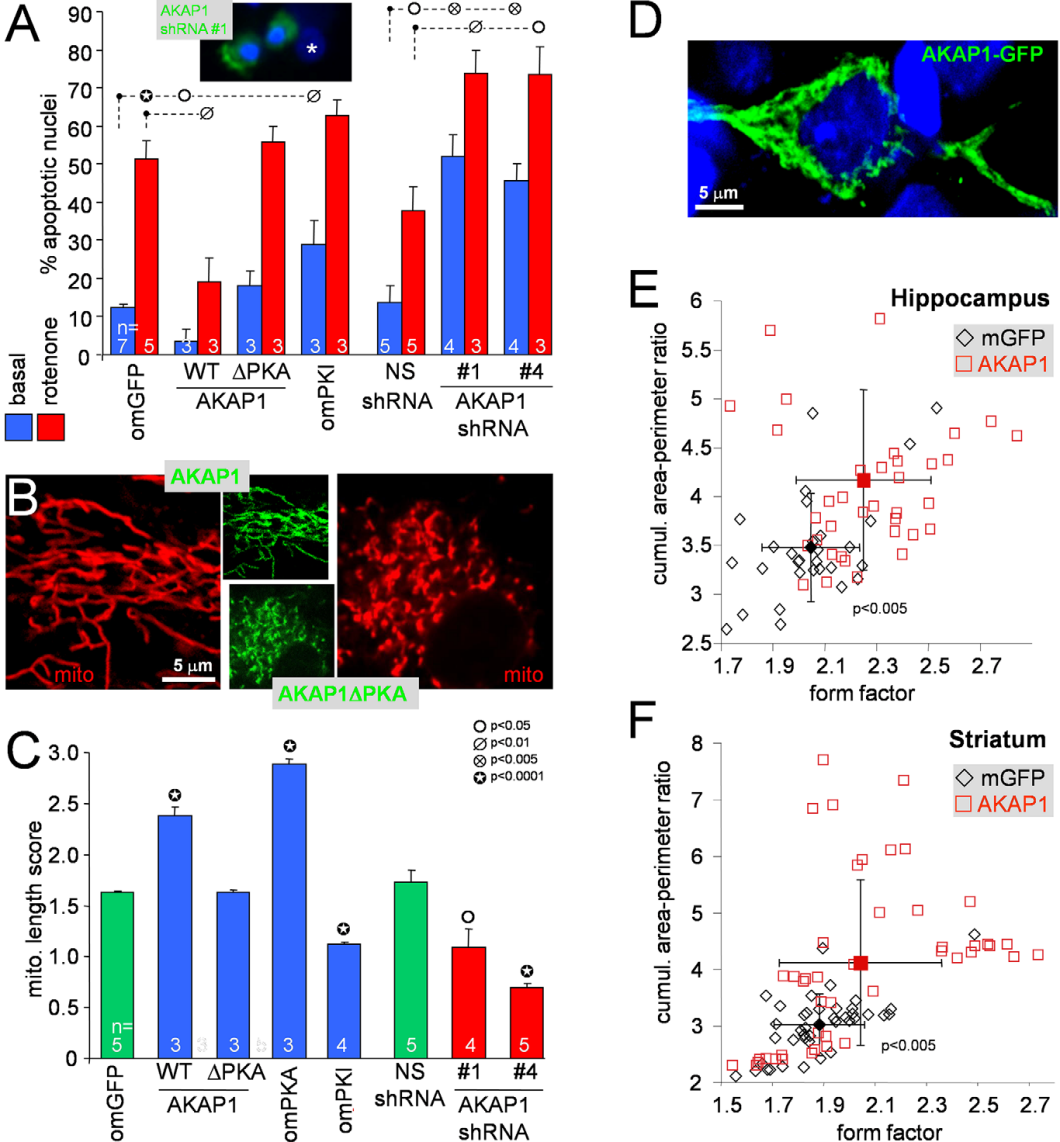
Mitochondrial PKA and AKAP1 promote neuronal survival and oppose mitochondrial fragmentation in vitro and in vivo. (A) Hippocampal neurons were transfected with the indicated cDNA and shRNA plasmids (AKAP1ΔPKA  =  I310P,L316P [PKA binding defective]; Figure S2C). After 3 d, cells were treated ± 400 nM rotenone for 2 d, fixed, and analyzed by counting apoptotic nuclei in the transfected neuron population (means ± s.e.m. of *n* = 3–7 experiments). The inset shows two transfected neurons (green) with apoptotic nuclei and an untransfected neuron with normal nucleus (asterisk). (B, C) Representative confocal sections of TMRM-stained (mito) hippocampal neurons (B) and their mitochondrial length scores (C) 3 d after transfection with the indicated cDNA and shRNA constructs are shown (means ± s.e.m. of *n* = 3–5 experiments; Student's *t* test comparisons between GFP fusion proteins and omGFP and between AKAP1 and NS shRNAs). (D–F) Rats injected with lentivirus expressing mitochondrial (m)GFP and AKAP1-GFP into the hippocampus and striatum of left and right hemispheres, respectively, were analyzed 7–14 d later for mitochondrial shape. Perfusion-fixed cryostat sections immunolabeled for GFP (representative confocal image in (D), counterstained for nuclei with TOPRO-3 [blue]) were subjected to ImageJ software-based morphometry. Scatter plots (E, F) correlate form factor (inverse of circularity of individual mitochondria) with cumulative area:perimeter ratio (a measure of network connectivity). Each open symbol represents average shape metrics from 10–22 z-sections of one neuron; filled symbols are population averages (± s.d., 29–42 neurons per condition from 2 (E) and 3 (F) rats).

AKAPs recruit the PKA holoenzyme to specific subcellular sites and substrates, which is critical for the physiological actions of the kinase [23]. Of the three AKAPs that have been localized to mitochondria, the OMM-anchored AKAP1 (also known as D-AKAP1, AKAP121, AKAP149) has previously been shown to enhance survival in PC12 cells [24]. We expressed AKAP1-GFP in hippocampal neurons and scored apoptosis either under basal conditions or 2 d after treatment with rotenone, an inhibitor of complex 1 of the electron transport chain that is commonly used as a chemical model of Parkinson's disease. Wild-type AKAP1, but not a point mutant that cannot bind the PKA holoenzyme (AKAP1ΔPKA =  I310P, L316P; Figure S2A), had a potent neuroprotective effect under both conditions (Figure 3A). Conversely, knockdown of endogenous AKAP1 with two different shRNAs (Figure S2B,C) dramatically amplified both basal and rotenone-induced neuronal death (Figure 3A). Additionally, as we observed in stressed PC12 cells, inhibiting outer mitochondrial PKA by expressing omPKI resulted in increased basal apoptosis in hippocampal neurons. Thus, AKAP1 enhances neuronal survival by recruiting PKA to the OMM. Our finding that AKAP1 and outer mitochondrial PKA are critical for neuronal viability contrasts with the mild phenotypes seen in unconditional AKAP1 knockout mice [25]. It is possible that other mitochondrial AKAPs may be able to compensate if AKAP1 is never expressed.

To investigate whether the pro-survival effects of PKA/AKAP1 are associated with changes in mitochondrial morphology, TMRM-stained mitochondria of hippocampal neurons were imaged 2–3 d after transfection. As previously reported in other cell types [26], AKAP1-GFP colocalized perfectly with mitochondria in hippocampal neurons (Figure 3B). Compared to OMM-targeted GFP or the AKAP1 mutant that cannot recruit PKA, overexpression of wild-type AKAP1 increased mitochondrial length scores in the somal mitochondria (Figure 3B,C). AKAP1 expression also increased mitochondrial form factor in dendrites of hippocampal neurons (Figure S3A,B). Targeting the PKA catalytic subunit directly to the OMM (omPKA) resulted in even more striking mitochondrial fusion, often culminating in perinuclear aggregation of mitochondria into a single mass. Conversely, either expression of OMM-tethered PKI or silencing of AKAP1 with two shRNAs induced significant somatic mitochondrial fragmentation as compared to OMM-targeted GFP and nonsense shRNA controls (Figure 3C). AKAP1 knockdown also induced mitochondrial fragmentation in dendrites of hippocampal neurons (Figure S3A,B). To rule out off-target effects, we silenced the endogenous protein in PC12 cells by co-expression of rat AKAP1-directed shRNAs with or without plasmids encoding human AKAP1, which diverges at both shRNA target sites. Human AKAP1 reversed the mitochondrial fragmentation induced by rat AKAP1-directed shRNAs (Figure S4A,B), indicating that loss of mitochondrial interconnectivity is due to specific silencing of the endogenous protein.

### AKAP1 Expression Leads to Mitochondrial Elongation In Vivo

Lentivirus expressing AKAP1-GFP was stereotaxically injected into the right striatum and hippocampus of rats; the left hemisphere received injections with a control lentivirus encoding mitochondrial (m)GFP (targeted via the N terminus of cytochrome oxidase VIII). After 7–14 d, mitochondrial shape was assessed by automated morphometry of confocal z-stacks from perfusion-fixed brain sections (Figure 3D). Compared to mGFP, lentiviral delivery of AKAP1 into the hippocampus and striatum induced robust mitochondrial fusion as indicated by two independent metrics, form factor, which measures elongation of individual mitochondria, and cumulative area:perimeter ratio, which measures mitochondrial interconnectivity (Figure 3E,F). Thus, neuroprotection by PKA/AKAP1 is associated with mitochondrial elongation both in vitro and in vivo.

### PKA-Induced Mitochondrial Remodeling Determines Neuronal Survival

**Figure 4:**
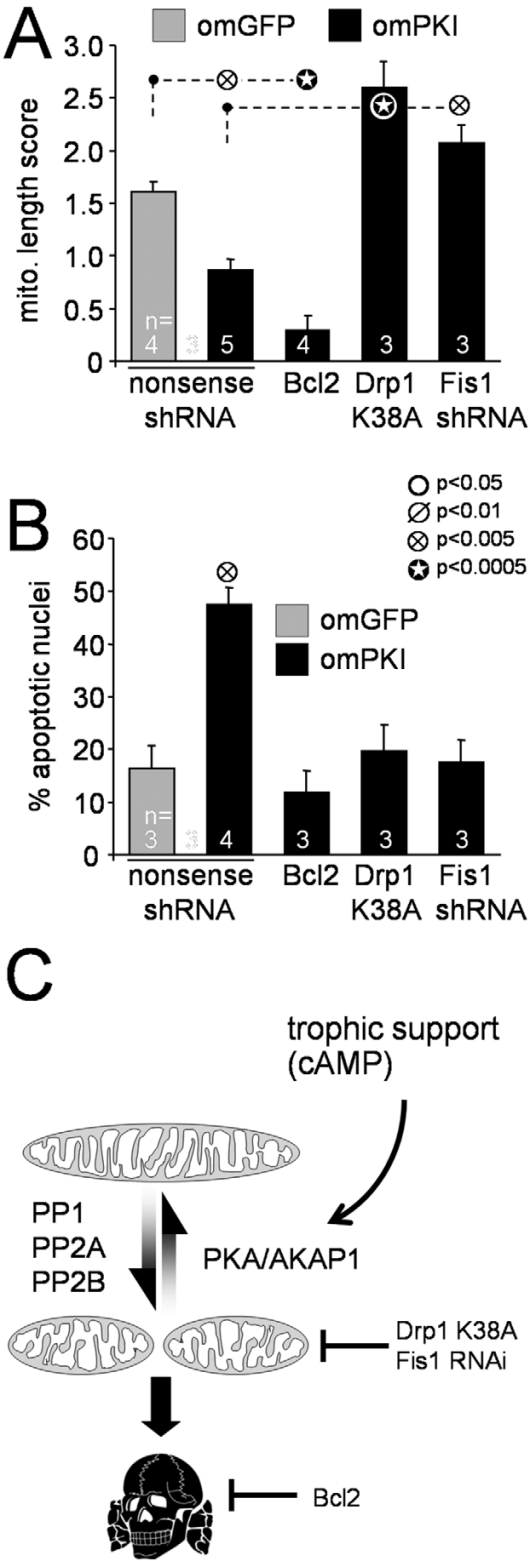
Mitochondrial restructuring underlies survival regulation by outer mitochondrial PKA. (A, B) Hippocampal neurons were transfected with the indicated plasmid combinations and scored after 2–3 d for mitochondrial morphology (A, live TMRM stain) or after 5–6 d for apoptosis (B, % transfected neurons with condensed/fragmented nuclei). Means ± s.e.m. of *n* = 3–6 experiments are shown. (C) Model summarizing effects of PKA/AKAP1 on mitochondrial shape and neuronal survival.

We carried out epistasis experiments in hippocampal neurons to determine whether PKA-mediated changes in mitochondrial shape and neuronal survival are causally related. Initially, we showed that overexpression of the antiapoptotic Bcl2 protein prevented apoptosis induced by inhibiting PKA with omPKI but had no effect on omPKI-induced mitochondrial fragmentation (Figure 4A,B). Similarly, Bcl2 rescued neurons from apoptosis due to AKAP1 silencing without restoring normal mitochondrial morphology (Figure S5). Therefore, mitochondrial fragmentation due to PKA inhibition occurs either independently of or upstream of apoptosis.

To distinguish between these possibilities, we interfered with mitochondrial fission by dominant-negative (K38A mutant) Drp1 or Fis1 knockdown. Blocking the mitochondrial fission machinery fully restored viability of neurons transfected with omPKI, demonstrating that PKA inhibition kills by inducing mitochondrial fission. In contrast, AKAP1 silencing with two shRNAs compromised neuronal viability in a manner that could not be reversed by inhibiting mitochondrial fission (Figure S5). Our data thus point to an essential function of AKAP1 beyond localizing PKA to maintain mitochondrial integrity. In this regard, the AKAP1 gene gives rise to multiple splice variants, which have been localized to the endoplasmatic reticulum and nuclear lamina, in addition to mitochondria. Also, AKAP1 interacts not only with PKA but also with various other signaling enzymes and mRNA [26].

The data thus far suggest a model in which the cAMP signaling through the outer mitochondrial PKA/AKAP1 complex opposes the mitochondria fragmenting activity of several protein phosphatases including PP2A [21], PP2B/calcineurin [18],[18], and likely PP1 (Figure 2C,D). Kinase/phosphates-regulated mitochondrial shape changes control neuronal vulnerability to a variety of challenges (Figure 4C).

### PKA/AKAP1 Slows Turnover of Mitochondrial Drp1

**Figure 5:**
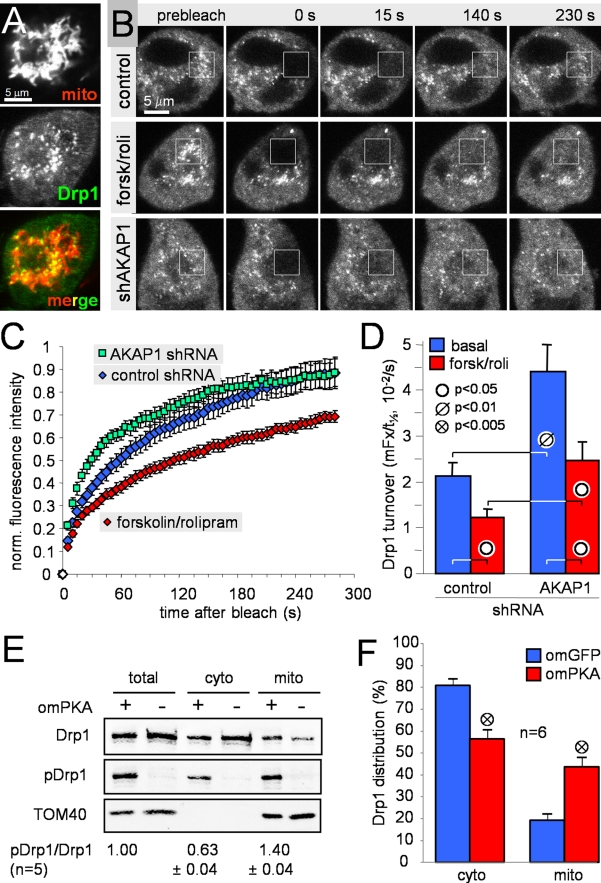
cAMP and PKA/AKAP1 decrease mobility and promote mitochondrial translocation of Drp1. (A) Confocal micrograph showing mixed cytosolic and mitochondrial localization of GFP-Drp1 in PC12 cells (mito, MitoTracker Deep Red). (B–D) FRAP analysis in PC12 cells shows opposite effects of PKA activation and AKAP1 knockdown on GFP-Drp1 dynamics. PC12 cells co-expressing GFP-Drp1 and either AKAP1-directed or control shRNA were treated ± forskolin/rolipram (25/1 µM, 1–3 h) and Drp1 turnover was measured by bleaching mitochondrial GFP-Drp1 in a 5×5 µm square and monitoring fluorescence recovery at 5 s intervals. (B) shows frames from representative cells (control, forsk/roli: control shRNA ± forskolin/rolipram; shAKAP1: AKAP1 shRNA #1), (C) shows averaged fluorescence recovery curves, and (D) plots Drp1 turnover as the ratio of mobile fraction (mFx) and 50% recovery time (t_1/2_) derived from biexponential fits (*R* ^2^∼0.99) of individual recovery curves (means ± s.e.m. of 8–10 cells for each condition from a representative experiment). (E–F) Subcellular fractionation of Drp1. COS cells co-expressing GFP-Drp1 with either outer mitochondrial (om) PKA (+) or omGFP (−) were permeabilized with digitonin (500 µg/ml) and fractionated into a cytosolic (cyto) and a heavy membrane fraction containing mitochondria (mito). Fractions were immunoblotted for total Drp1, phospho-Ser_PKA_ Drp1 (pDrp1), and the mitochondrial marker TOM40 and analyzed by densitometry (E, representative blot; F, summary showing means ± s.e.m. of 6 independent experiments).

PKA/AKAP1 could maintain elongated mitochondria by inhibiting fission or promoting fusion reactions. The mitochondrial fission enzyme Drp1 was previously shown to be phosphorylated by PKA and phospho-mimetic substitution of the targeted Ser residue causes mitochondrial elongation [18],[19]. We therefore examined the mobility of Drp1 by fluorescence recovery after photobleaching (FRAP), replacing endogenous Drp1 in PC12 cells with the GFP-tagged protein by a single-plasmid RNAi/expression strategy (Figure S2D) [18]. Similar to the endogenous fission enzyme, GFP-Drp1 was localized diffusely in the cytosol, as well as in punctate structures on mitochondria (Figure 5A). Fluorescence recovery of mitochondrial GFP-Drp1 could be well approximated by double exponential fits (*R* ^2^>0.99) and was largely independent of the size of the bleached area, indicating that Drp1 mobility follows reaction- rather than diffusion-limited kinetics [27]. Drp1 fluorescence recovery was also shown to be unaffected by prior fragmentation of mitochondria via protonophore treatment (Figure S6), indicating that Drp1 turnover determines mitochondrial shape and not vice versa.

Silencing of AKAP1 increased the cytosolic pool at the expense of the mitochondrial pool of GFP-Drp1 and accelerated fluorescence recovery of mitochondrial GFP-Drp1. Activation of PKA by forskolin/rolipram treatment had the opposite effect, increasing mitochondrial localization of GFP-Drp1 and slowing its turnover (Figure 5B,C). Faster Drp1 recovery was reflected in an increase in the plateau of the double exponential fit (mobile fraction) and a decrease in the 50% recovery time constant (t_1/2_); therefore, we expressed Drp1 turnover as the ratio of the two curve-fitting parameters (Figure 5D).

In support of the imaging experiments, subcellular fractionation of GFP-Drp1 expressed in COS cells showed that 80% of the fission protein is cytosolic, while 20% localizes to a heavy membrane fraction that includes mitochondria (Figure 5E,F). Co-expression of OMM-targeted PKA catalytic subunit induced phosphorylation of Drp1 Ser_PKA_ mainly in the mitochondrial fraction as detected with a phosphorylation state-specific antibody. Moreover, omPKA expression resulted in a robust relocalization of Drp1 towards mitochondria (43%, Figure 5E,F). Together, these data implicate Drp1 as a target of PKA/AKAP1 and suggest that phosphorylation may slow the catalytic cycle of Drp1 by trapping the fission protein at the mitochondrial membrane.

### Drp1 Ser_PKA_ Phosphorylation Is Required for PKA/AKAP1-Mediated Mitochondrial Fusion

**Figure 6:**
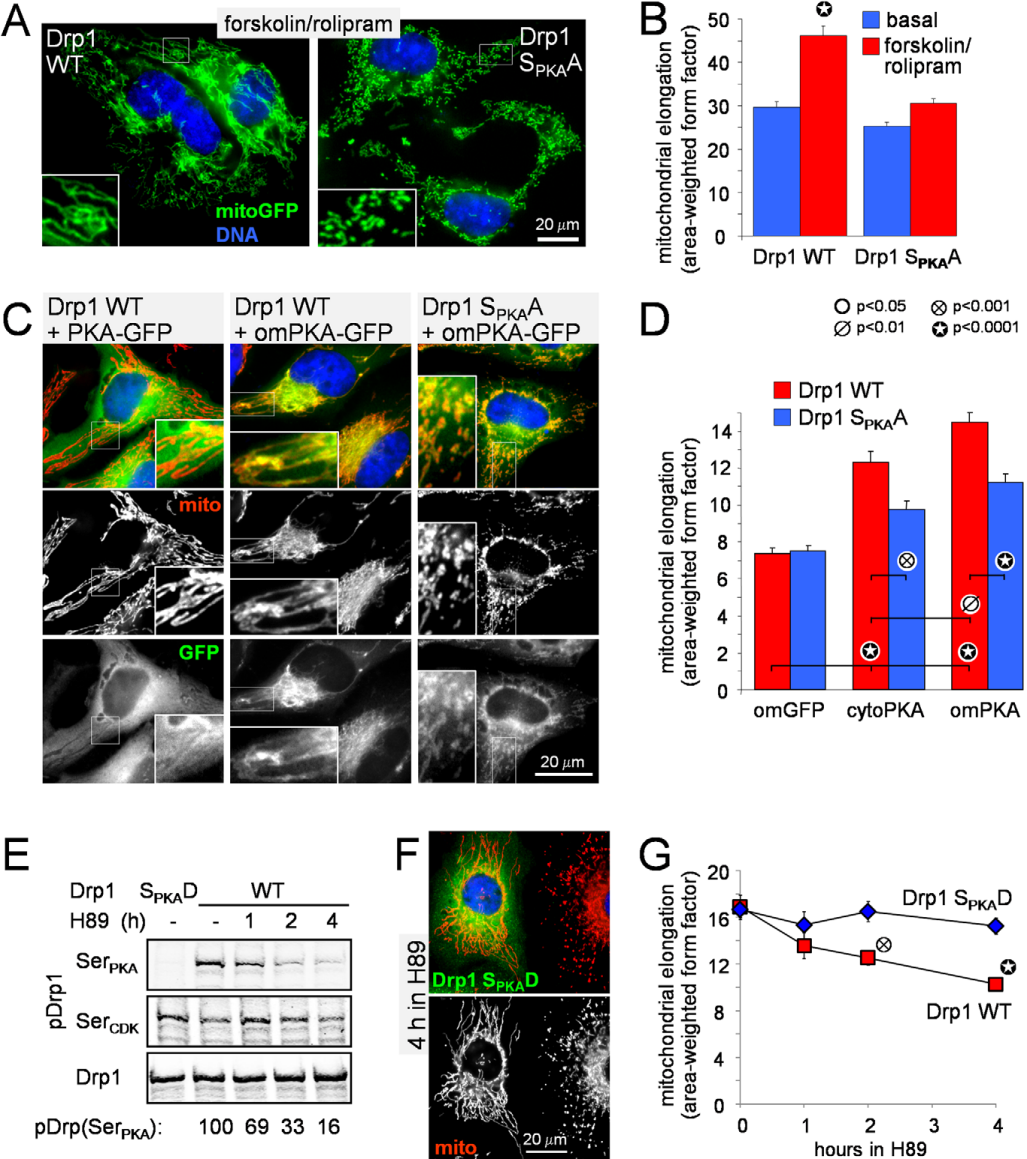
PKA shapes mitochondria through phosphorylation of Drp1 at Ser_PKA_. (A, B) PC12 cells expressing mitochondrial GFP (green) and either wild-type or S_PKA_A-mutant Drp1 instead of endogenous Drp1 were treated ± forskolin/rolipram (25/2 µM, 3 h), fixed, and epifluorescence micrographs (representatives in A) were subjected to digital morphometry (means ± s.e.m. of ∼300 cells per condition from a representative experiment). (C–D) HeLa cells co-expressing the indicated constructs (om, outer mitochondrial) were fixed and processed for immunofluorescence for mitochondrial cytochrome oxidase II (mito, red) and GFP (green). Shown are representative images (C) and mitochondrial morphology analysis (D, means ± s.e.m. of ∼200–300 cells per condition from a representative experiment). (E–G) HeLa cells expressing WT or S_PKA_D-mutant GFP-Drp1 were incubated for up to 4 h with the PKA inhibitor H89 (20 µM) and analyzed by quantitative immunoblotting for phosphorylated (pDrp1, Ser_PKA_, and Ser_CDK_) and total Drp1 (E) or by immunofluorescence for mitochondrial morphology (F, representative image; G, means ± s.e.m. of ∼200 cells/condition from a representative experiment). For immunoblot analysis only (E), trace amounts (5% plasmid) of PKA catalytic subunit were cotransfected to increase the signal strength with the phospho-Ser_PKA_ Drp1 antibody.

To explore the role of Drp1 phosphorylation in mitochondrial remodeling by PKA, we replaced endogenous Drp1 in PC12 cells with mutant Drp1 that cannot be phosphorylated by PKA (Ser_PKA_Ala, Ser_PKA_ =  Ser656 in rat splice variant 1) and analyzed mitochondrial morphology at basal or forskolin/rolipram-stimulated cAMP levels. Mutation of Ser_PKA_ in Drp1 rendered cells largely refractory to mitochondrial elongation by cAMP (Figure 6A,B). To confirm the critical importance of Drp1 Ser_PKA_ in the mitochondria-shaping effects of PKA in non-neuronal cells, HeLa cells expressing either wild-type or S_PKA_A-mutant Drp1 were also transfected with the catalytic subunit of PKA, either with or without an OMM targeting sequence. Compared to control transfected cells, both forms of PKA resulted in significant mitochondrial elongation, with mitochondrial PKA being more effective than cytosolic PKA. The non-phosphorylatable Drp1 mutant attenuated mitochondrial remodeling by either form of PKA to a similar extent (Figure 6C,D).

We then explored the role of basal PKA activity in the maintenance of mitochondrial shape. Incubation of HeLa cells with the PKA inhibitor H89 resulted in a protracted loss of phosphate from Drp1 Ser_PKA_ but did not affect Drp1 phosphorylation at the neighboring Ser_CDK_ (Figure 6E). Paralleling the time course of Drp1 Ser_PKA_ dephosphorylation, mitochondria in wild-type GFP-Drp1-expressing cells underwent fragmentation upon endogenous PKA inhibition. HeLa cells expressing pseudophosphorylated Drp1 (Ser_PKA_Asp mutant) on the other hand were completely refractory to H89-induced mitochondrial fission (Figure 6F,G).

**Figure 7:**
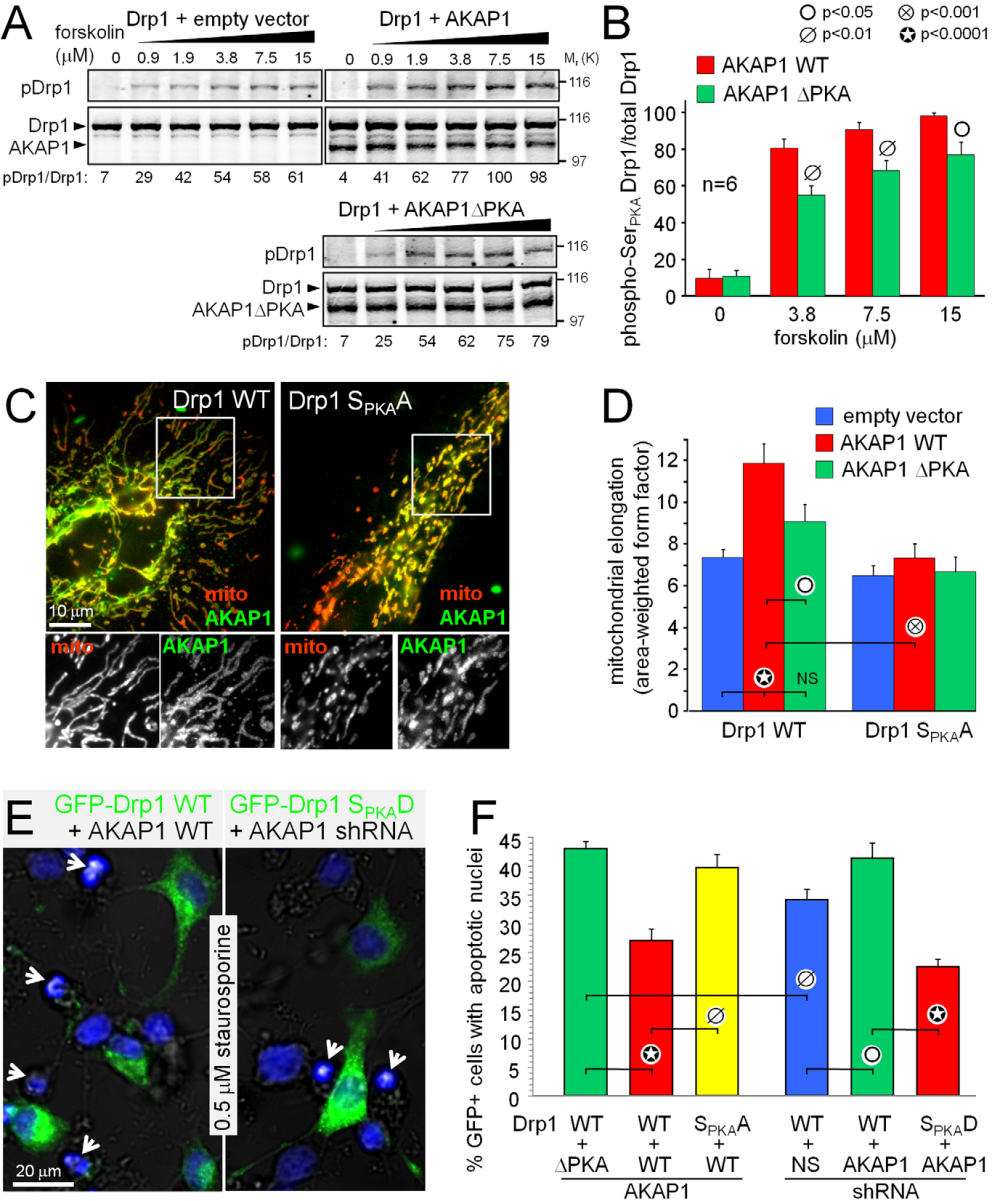
AKAP1 promotes mitochondrial elongation and neuronal survival by enhancing phosphorylation of Drp1 at Ser_PKA_. (A, B) COS cells co-expressing GFP-Drp1 and either empty vector, wild-type, or ΔPKA-mutant GFP-AKAP1_1–524_ were stimulated for 45 min with increasing concentrations of forskolin/rolipram (rolipram at 1/30^th^ of the indicated forskolin concentrations), and total cell lysates were probed for phospho-Ser_PKA_ Drp1 and GFP (detecting Drp1 and AKAP1) on the same blot using a dual-channel infrared imager. Drp1 phosphorylation was quantified as the ratio of phospho- to total Drp1 signals normalized to the highest value and is shown as part of the representative blots (A) and the summary graph (B, means ± s.e.m. of 6 independent experiments). (C, D) HeLa cells co-expressing the indicated constructs (ΔPKA  =  I310P, L316P-mutant) were treated with forskolin/rolipram (25/2 µM, 3 h) and processed for immunofluorescence for mitochondrial cytochrome oxidase II (mito, red) and V5-tagged AKAP1 (green). Shown are representative images (C) and mitochondrial morphology analysis (D, mean ± s.e.m. of ∼200–300 cells per condition from a representative experiment). (E, F) PC12 cells were cotransfected with GFP-Drp1/shRNA expression plasmids and either AKAP1 cDNAs or shRNAs. Cultures were treated 48 h posttransfection with 0.5 µM staurosporine for 24 h, fixed, and stained with Hoechst 33342. Representative images in (E) show overlays of cell contours (bright field), GFP-Drp1 (green), and Hoechst-stained nuclei (blue), with transfected cells displaying normal nuclear morphology and many untransfected cells having condensed, apoptotic nuclei (arrows). Apoptosis was quantified as the percentage of GFP-positive cells with condensed or fragmented nuclei (E, means ± s.e.m. of 6–13 image fields with ∼300 transfected cells per condition from one experiment representative of three).

To examine the influence of AKAP1 on Drp1 phosphorylation, COS cells were co-transfected with Drp1 and either empty vector, wild-type AKAP1, or the PKA-binding deficient AKAP1 mutant and stimulated with forskolin/rolipram. Especially at lower forskolin/rolipram concentrations, recruitment of the PKA holoenzyme to mitochondria via wild-type AKAP1 significantly enhanced Drp1 phosphorylation at Ser_PKA_ (Figure 7A,B). Essentially identical results were obtained with neuronal PC12 cells (Figure S7). Rationalizing these findings, we hypothesize that Ser_PKA_ of mitochondrial Drp1 is more exposed to phosphorylation by PKA; alternatively, Ser_PKA_-phosphorylated, mitochondrial Drp1 may be relatively protected from cytosolic protein phosphatases, such as calcineurin.

Next, we investigated the role of Drp1 phosphorylation in AKAP1-mediated mitochondrial fusion. For this set of experiments, HeLa cells expressing empty vector, wild-type, or PKA binding-deficient AKAP1 in addition to wild-type or S_PKA_A-mutant Drp1 were treated with forskolin/rolipram to activate PKA. As in neuronal cells, AKAP1 promoted mitochondrial network formation that depended on recruitment of PKA.

Furthermore, PKA/AKAP1-induced mitochondrial elongation was nearly abolished when endogenous Drp1 was replaced with the Ser→Ala mutant (Figure 7C,D).

We confirmed an epistatic relationship between AKAP1 and Drp1 Ser_PKA_ in primary hippocampal neurons. Substitution of endogenous with Ser→Ala-mutant Drp1 resulted in mitochondrial fragmentation, whereas the Ser→Asp mutant caused mitochondrial elongation as assessed in dendrites (form factor: Figure S8B; length: Figure S8C). Co-expression of wild-type AKAP1 lengthened dendritic mitochondria in wild-type GFP-Drp1 substituted neurons, but not in neurons expressing either Ser_PKA_ mutant. Neurons were unresponsive to AKAP1 ΔPKA transfection (Figure S8). These experiments indicate that PKA/AKAP1 inhibits mitochondrial fission by phosphorylating Drp1 at Ser_PKA_.

AKAP1 has previously been reported to promote phosphorylation of the proapoptotic Bcl2 family member Bad in PC12 cells, and Bad phosphorylation and cytosolic sequestration was proposed to underlie the neuroprotective effect of PKA/AKAP1 [24]. To examine whether Drp1 may instead be the critical prosurvival substrate of PKA/AKAP1, we challenged transiently transfected PC12 cells with the classical apoptosis inducer staurosporine, a broad-spectrum kinase inhibitor previously shown to dephosphorylate Drp1 at Ser_PKA_ [18]. Compared to AKAP1 ΔPKA or scrambled shRNA, overexpression of wild-type AKAP1 significantly attenuated apoptosis induced by staurosporine (0.5 µM for 24 h) when endogenous Drp1 was replaced with wild-type GFP-Drp1. Replacement with Ser→Ala mutant Drp1, instead, negated the antiapoptotic effect of AKAP1 (Figure 7E,F). Conversely, AKAP1 knockdown in wild-type GFP-Drp1 expressing cells moderately increased the percentage with apoptotic nuclei compared to scrambled shRNA. Pseudophosphorylated (S→D mutant) Drp1 overcame the proapoptotic effect of AKAP1 shRNA, reducing staurosporine-induced apoptosis to levels comparable to AKAP1 overexpression (Figure 7E,F). These results provide strong evidence that AKAP1 acts through Drp1 (and not Bad) to inhibit the intrinsic apoptosis cascade in PC12 cells.

### Ser_PKA_ Phosphorylation by PKA/AKAP1 Assembles Drp1 Into Large, Slowly Recycling Mitochondrial Complexes

**Figure 8:**
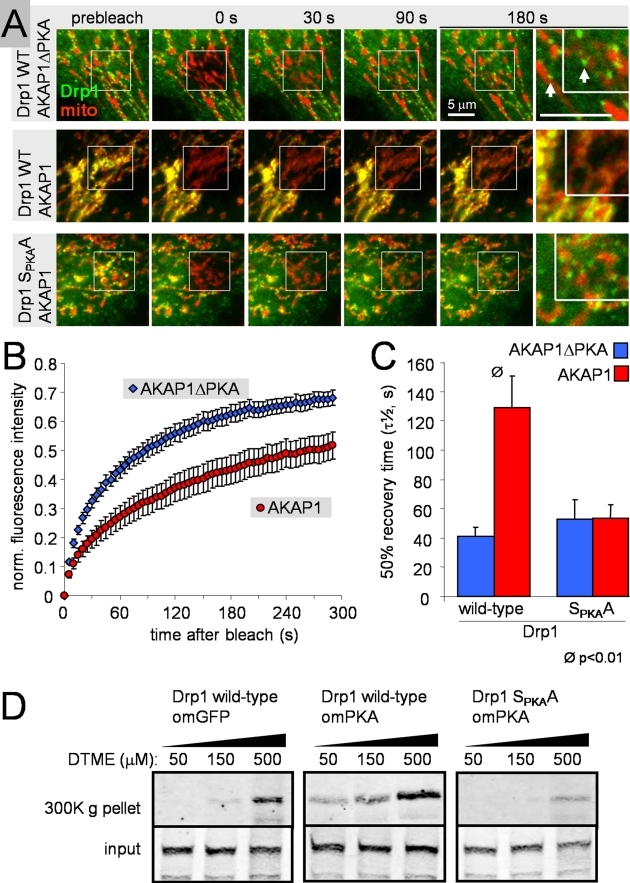
PKA/AKAP1 recruits Drp1 into large, slow turnover complexes by phosphorylating Ser_PKA_. (A–C) HeLa cells co-expressing wild-type or S_PKA_A-mutant GFP-Drp1 and either wild-type or PKA-binding deficient (ΔPKA) AKAP1 were subjected to FRAP analysis. (A) Frames showing time lapse series of representative cells (green: GFP-Drp1, red: MitoTracker Deep Red) with enlargements of the 180 s frame demonstrating recovery of Drp1 into mitochondrial foci (arrows). (B) Average recovery curves of cells expressing wild-type GFP-Drp1 and either wild-type or mutant AKAP1, and (C) plots mitochondrial Drp1 turnover as the 50% recovery time calculated from biexponential fits of individual recovery curves (*R* ^2^∼0.992; means ± s.e.m. of 5–9 cells each from a representative experiment). (D) COS cells cotransfected with Drp1 and either omGFP or omPKA were treated for 5 min with the indicated concentration of the reversible, membrane permeant crosslinker dithiobismaleimidoethane (DTME), and cleared cell lysates were subjected to ultracentrifugation to sediment Drp1 complexes.

FRAP and subcellular fractionation experiments so far suggest that AKAP1 recruits PKA to phosphorylate Drp1 at the OMM, which inhibits the membrane scission activity of Drp1 by trapping the protein in slow-turnover complexes (Figure 5). We returned to FRAP experiments in HeLa cells to establish a requirement for PKA binding to AKAP1 and Ser_PKA_ phosphorylation of Drp1 in this process. Compared to AKAP1ΔPKA, co-expression of wild-type AKAP1 significantly slowed fluorescence recovery of mitochondrial GFP-Drp1 (>3-fold increase in t_1/2_; Figure 8A–C, Video S1). Furthermore, as seen in forskolin/rolipram-stimulated PC12 cells, wild-type AKAP1 expression resulted in a shift of GFP-Drp1 localization from cytosol to mitochondria (Figure 8A). In striking contrast, PKA site-mutant (S_PKA_A) GFP-Drp1 was completely insensitive to PKA/AKAP1 recruitment, displaying rapid recovery comparable to wild-type Drp1 cotransfected with AKAP1ΔPKA (Figure 8A,C).

Cytosolic Drp1 is a tetramer, which upon translocation to the OMM is thought to oligomerize into spiral or ring-shaped superstructures. In analogy to dynamin, GTP hydrolysis may trigger disassembly of Drp1 oligomers and concomitant membrane fission [28]. In order to provide evidence that outer mitochondrial PKA promotes Ser_PKA_-dependent oligomerization of Drp1, we performed crosslinking experiments with dithiobismaleimidoethane (DTME), a membrane-permeant, reversible, and Cys-reactive crosslinker. Transfected COS cells were crosslinked in vivo, and cell lysates were subjected to ultracentrifugation (300,000 x g) to assess Drp1 assembly into large, sedimentable complexes. Compared to co-expression of OMM-targeted GFP, OMM-targeted PKA enhanced oligomerization of wild-type but not Ser_PKA_-mutant Drp1 (Figure 8D), providing a biochemical correlate for the slowly recycling, mitochondrial Drp1 pool we observed in our FRAP experiments.

### GTPase-Impairment Phenocopies Phosphorylation of Drp1

How does PKA/AKAP1-mediated phosphorylation stabilize Drp1 oligomers at the OMM? Ser_PKA_ phosphorylation was previously shown to inhibit intrinsic GTP hydrolysis by Drp1 [19]. Dnm1, the yeast ortholog of Drp1, assembles into fission-competent complexes in its GTP-bound state, but assembly also stimulates GTP hydrolysis, effectively limiting the size of Dnm1 oligomers at the OMM [28]. Therefore, an attractively simple hypothesis compatible with our observations is that PKA/AKAP1 phosphorylation of Drp1 attenuates GTP hydrolysis, thereby allowing Drp1 oligomers to grow beyond a size that is compatible with membrane remodeling.

**Figure 9:**
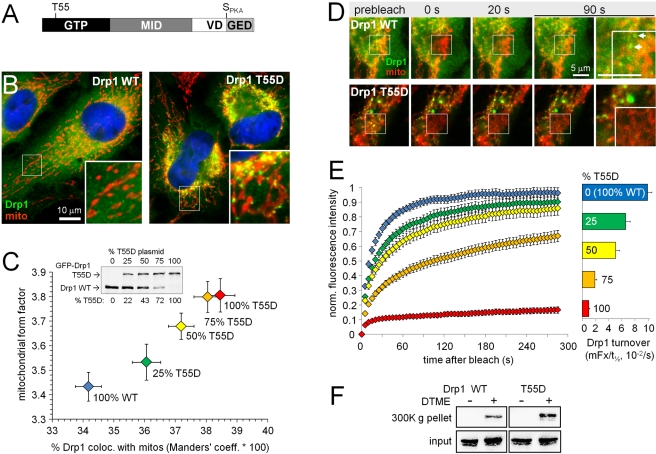
A GTPase-compromised Drp1 mutant accumulates in slowly recycling complexes at mitochondria. (A) Drp1 domain diagram. (B–C) Endogenous Drp1 in HeLa cells was replaced by transfection of the indicated ratios of wild-type (WT) and T55D mutant GFP-Drp1 expression plasmids. Cells were fixed and analyzed for mitochondrial length and Drp1 colocalization with mitochondria using ImageJ software [22],[61]. (B) Representative images showing punctate localization of GFP-Drp1 T55D (green) on mitochondria (cytochrome oxidase subunit II antibody). (C) Correlation between mitochondrial length and mitochondrial localization of Drp1 as a function of Drp1 T55D expression (means ± s.e.m. of ∼200 cells per condition from a representative experiment). Wild-type and T55D-mutant Drp1 expression levels are similar and scale with plasmid amounts (inset). (D, E) Turnover of GFP-Drp1 WT and T55D was analyzed by FRAP as in Figure 8. (D) Frames showing representative time lapse series (green: GFP-Drp1, red: MitoTracker Deep Red, see Video S2) of HeLa cells expressing 100% WT or T55D Drp1, with the last frame expanded to show recovery of WT Drp1 into mitochondrial foci (arrows). (E) Average recovery curves (left) and curve fit-derived turnover (right, ratio of mobile fraction [mFx] and 50% recovery time [t_1/2_]) from cells expressing varying ratios of WT and T55D-mutant GFP-Drp1 (mean ± s.e.m. of 12–20 cells each from a representative experiment). (F) COS cells expressing GFP-Drp1 WT or T55D were incubated with DTME (5 min, 500 µM), and cleared cell lysates were subjected to ultracentrifugation to sediment Drp1 complexes (∼2-fold increase with the T55D mutation).

This hypothesis predicts that GTPase-impaired Drp1 mutants should behave similarly to phospho-Ser_PKA_ Drp1. We initially analyzed the widely used dominant-negative K38A mutant of Drp1, which affects a critical Lys residue in the nucleotide binding fold. However, Drp1 K38A was found to localize exclusively in large spherical aggregates that only occasionally overlapped with mitochondria and that did not exchange with one another as detectable by FRAP analysis (unpublished data). Aiming for a milder phenotype, we targeted Thr55 in the Drp1 GTPase domain (Figure 9A) based on a mutagenesis study of the related dynamin-2. Ser61, the corresponding residue in dynamin-2, lies within the nucleotide binding pocket but does not directly coordinate GTP [29]. Mutation of Ser61 to Asp was shown to significantly lower the maximal rate of GTP hydrolysis (K_cat_) without affecting the K_m_ or apparent affinity of dynamin-2 for GTP [30].

To assess the effect of the T55D mutation on mitochondrial morphology, HeLa cells were transfected with different ratios of wild-type and T55D-mutant GFP-Drp1 plasmid (replacing endogenous Drp1 by RNAi from the same plasmid). Drp1 T55D expression resulted in a dose-dependent increase in mitochondrial length, demonstrating that the mutation impairs Drp1's fission activity (Figure 9B,C). Remarkably, GFP-Drp1 T55D also displayed a more pronounced, punctate mitochondrial localization than wild-type Drp1 (Figure 9B). Quantitative analysis confirmed that GFP-Drp1 colocalization with mitochondria (Manders' coefficient) was positively correlated with relative expression levels of Drp1 T55D, as well as with mitochondrial length (Figure 9C). Further FRAP analysis demonstrated that the T55D substitution dramatically attenuates mitochondrial Drp1 dynamics, again in a dose-dependent manner, slowing turnover by up to 10-fold (Figure 9D,E; Video S2). Similar to phosphorylation at Ser_PKA_, the T55D mutation also increased the propensity of Drp1 to form sedimentable oligomeric structures after intact cell crosslinking (Figure 9F).

Therefore, inhibition of GTP hydrolysis (either by mutation of the GTPase domain or by phosphorylation of Ser_PKA_ by PKA/AKAP1) leads to a stable accumulation of Drp1 at mitochondria, likely by interfering with a disassembly step that is required for mitochondrial fission.

### PKA Phosphorylation and T55D Substitution Extend the Lifetime of Mitochondrial Drp1 Foci

**Figure 10:**
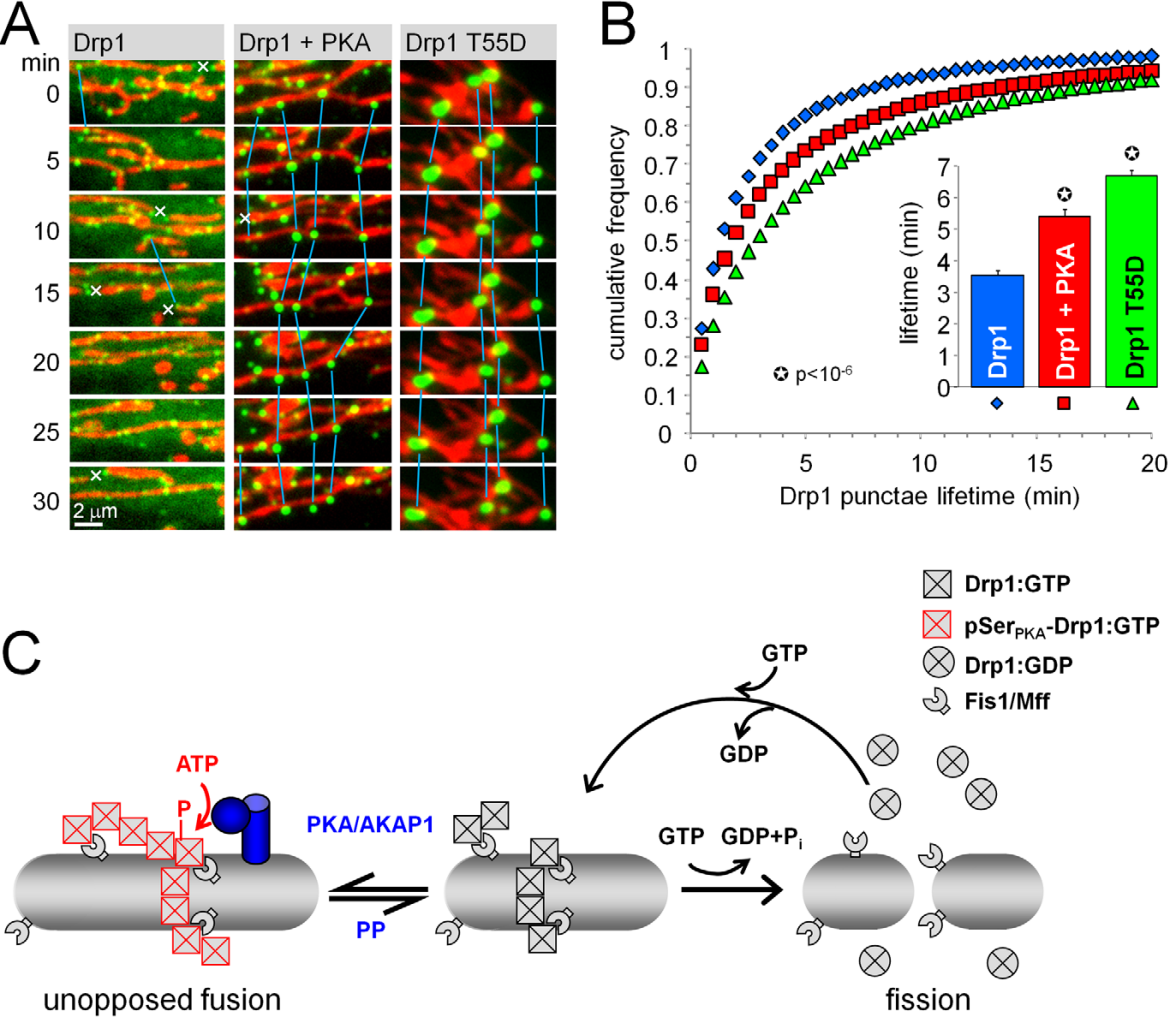
PKA phosphorylation and T55D mutation prolong the lifetime of mitochondrial Drp1 foci. (A, B) HeLa cells transfected with GFP-Drp1 (green), dsRed2/mito (COX8 matrix targeting sequence, red), ± PKA catalytic subunit were imaged for ≥1 h at 37°C, capturing images every 30 s. (A) Representative frames of time lapse series (see Video S3). Blue lines connect GFP-Drp1 punctae that could be tracked for at least 5 min; x symbols denote mitochondrial fission events. Note that GFP-Drp1 punctae often split with the fragmenting mitochondrion. (B) Cumulative frequency plot of Drp1 punctae lifetimes and average lifetimes (bar graph inset; means ± s.e.m. of 35–62 cells and 11,000 to 40,000 punctae per condition from two independent experiments). (C) Model of mitochondrial fusion by PKA/AKAP1. GTP-bound Drp1 translocates to mitochondria to assemble into oligomeric complexes. Drp1 assembly stimulates GTP hydrolysis, leading to mitochondrial fission and release of Drp1 into the cytosol to complete the cycle. OMM-anchored PKA/AKAP1 phosphorylates Drp1 at Ser_PKA_, stabilizing the GTP-bound state to promote growth of Drp1 complexes to a size that is incompatible with membrane scission. Protein phosphatases (PP), including calcineurin, dephosphorylate Drp1 Ser_PKA_ to return Drp1 into its active, rapidly cycling state.

In an effort to provide direct evidence that PKA phosphorylation slows the turnover of mitochondrial Drp1 complexes, we imaged HeLa cells expressing GFP-Drp1 and mitochondrial dsRed2 at high magnification (630×). Wild-type GFP-Drp1 foci were mostly found to abut mitochondria, seemingly randomly translocating along their length and pivoting around the organelle (Figure 10A, Video S3). Mitochondrial fission events were observed only at sites of GFP-Drp1 accumulation, and GFP-Drp1 punctae frequently divided to segregate with the new mitochondrial ends. When PKA was co-expressed, GFP-Drp1 punctae appeared larger and less dynamic, and the frequency of mitochondrial fragmentation events decreased. Foci formed by GTPase-impaired, T55D mutant Drp1 displayed a similar albeit even more accentuated behavior, which correlated with the near absence of fission events during the 1 h recording period (Figure 10A, Video S3).

Movies were subjected to automated particle tracking analysis [31], extracting average lifetimes of mitochondria-associated GFP-Drp1 punctae at 37°C. Wild-type Drp1 punctae could be tracked for an average of 3.5 min, whereas co-expression of PKA or the T55D mutation increased the persistence of GFP-Drp1 punctae to 5.4 and 6.7 min, respectively (Figure 10B).

## Discussion

This report establishes a role for outer mitochondrial PKA and, in particular, the PKA/AKAP1 complex in the maintenance of mitochondrial integrity and the protection from neuronal injury. The importance of cAMP/PKA signaling in cell survival is well documented [26]. Phosphorylation and inactivation of Bad, a pro-apoptotic Bcl2-family protein, has been put forward as one of the critical survival promoting substrates of mitochondria-localized PKA [32],[33]. While AKAP1 expression in PC12 cells was shown to increase Bad phosphorylation at multiple sites [24], our results indicate that Bad phosphorylation does not significantly contribute to the anti-apoptotic function of AKAP1. Instead, we present evidence that PKA targeting via AKAP1 opposes cell death mainly by gating Drp1-dependent mitochondrial fission. Specifically, inhibition of endogenous PKA via OMM-targeted PKI leads to mitochondrial fragmentation and sensitizes neurons to pro-apoptotic stimuli, both of which are reversed by blocking the mitochondrial fission machinery. Conversely, recruiting PKA to mitochondria via expression of AKAP1 resulted in mitochondrial elongation in cell culture and in vivo and protected hippocampal neurons from rotenone toxicity. Both mitochondrial elongation and survival enhancement by AKAP1 required PKA anchoring and Ser_PKA_-phosphorylatable Drp1, indicating a critical role for the PKA-Drp1 axis.

However, our results do not rule out the possibility that PKA may cause mitochondrial elongation by promoting mitochondrial fusion events in addition to inhibiting mitochondrial division. For instance, a recent report showed that forskolin can stimulate mitochondrial fusion in a cell-free assay [34].

AKAP1 is a large, multifunctional adaptor protein with several splice variants and a highly conserved N-terminal transmembrane domain that acts as a mitochondrial targeting sequence [35]. Besides localizing the PKA holoenzyme, AKAP1 also interacts with the tyrosine phosphatase PTPD1, and through PTPD1 with the tyrosine kinase Src, as well as two Ser/Thr phosphatases, PP1 and PP2B [36]–[38]. The C-terminus of AKAP1 contains Tudor and KH RNA binding domains, which were suggested to localize nucleus-derived mRNAs encoding mitochondrial proteins close to their destination [39].

A recent study described nuclear aggregation of mitochondria upon overexpressing an N-terminal fragment of AKAP1 containing the PKA binding domain. The authors did not, however, investigate whether this effect was PKA dependent and instead attributed the phenotype to a lack of RNA binding to the missing C terminus [37]. In the present study, we consistently observed elongation but rarely nuclear aggregation of mitochondria, regardless of whether full-length or C-terminally truncated AKAP1 (residues 1–524) was expressed. High levels of AKAP1 overexpression did sometimes induce nuclear aggregation of mitochondria, which may therefore be secondary to exaggerated fusion of the organelle. Mitochondrial remodeling depended on an intact PKA binding domain and was phenocopied and reversed by direct OMM tethering of PKA and PKI, respectively. Thus, PKA targeting is both necessary and sufficient for AKAP1-dependent regulation of mitochondrial morphogenesis.

Both the PKA and the PTPD1/Src interaction domains are important for maintenance of mitochondrial membrane potential by AKAP1 [40]. Hence, either mitochondrial recruitment of PTPD1/Src or an as yet undefined structural role of AKAP1 may explain why mitochondrial fission inhibitors rescue hippocampal neurons from omPKI expression but not from AKAP1 knockdown.

Consistent with an essential role for AKAP1 in neuronal survival, a recent report demonstrated that ischemia induces expression of the E3 ubiquitin ligase Seven In-Absentia Homolog 2 (Siah2), which targets AKAP1 for rapid proteasomal degradation [41]. Our study predicts that the hypoxia-induced loss of PKA anchoring at the OMM leads to disinhibition of Drp1 and contributes to the massive mitochondrial fragmentation that is a hallmark of ischemic brain injury [42].

We have identified a conserved PKA phosphorylation site in the GTPase effector domain of Drp1 as the principal mediator of PKA/AKAP1-induced mitochondrial remodeling. AKAP1-mediated redistribution of PKA was shown to augment Drp1 Ser_PKA_ phosphorylation, and mitochondria of cells expressing Ser_PKA_Ala-substituted Drp1 were unresponsive to cAMP and PKA/AKAP1. As to a mechanism, Drp1 activation via AKAP1 silencing was associated with accelerated cycling of the fission enzyme between cytosolic and mitochondrial pools. Conversely, Drp1 inhibition via cAMP or PKA recruitment or overexpression resulted in the accumulation of stable Drp1 oligomers at mitochondria and in an extension of the lifetime of mitochondrial Drp1 foci. Because Ser_PKA_ phosphorylation decreased the K_cat_ of GTP hydrolysis and because a mutation that stabilizes the GTP bound form of Drp1 mimicked the effects of PKA phosphorylation on localization and dynamics of the fission enzyme, modulation of Drp1's GTP cycle emerges as a probable mechanism for the mitochondria-stabilizing and neuroprotective actions of PKA/AKAP1.

A previous FRAP study demonstrated that the apoptosis inducer staurosporine causes accumulation of slowly recycling mitochondrial YFP-Drp1 complexes, which colocalize with Bax and Bak. Since the arrest of Drp1 cycling occurs after mitochondria have fragmented but before they release cytochrome C, this phenomenon may be related to the proapoptotic christae remodeling activity of Drp1 [43]. Given that the pan-kinase inhibitor staurosporine actually inhibits Drp1 phosphorylation at Ser_PKA_ [18], mitochondrial accumulation of Drp1 during apoptosis likely occurs by a mechanism distinct from the one reported here, such as Drp1 sumoylation ([43],[44], but see [45]).

Seemingly at odds with our findings, another study previously suggested that calcineurin-mediated dephosphorylation of Drp1 at Ser_PKA_ promotes translocation of the fission enzyme to mitochondria, a conclusion largely based on overexpression of phosphorylation site-mutant Drp1 [17]. Confirming and extending the findings of that report, we found that pseudophosphorylated (S_PKA_D-mutant) GFP-Drp1 partitions mostly with the cytosolic fraction (>90%), oligomerizes less readily than wild-type Drp1, and only infrequently forms mitochondrial punctae (unpublished data), which is essentially opposite to the phenotype of Drp1 phosphorylated by PKA. Because Asp substitution of Drp1 Ser_PKA_ at most incompletely reproduces the inhibitory effect of Ser_PKA_ phosphorylation on in vitro GTP hydrolysis [17]–[19], we propose that the supposedly phosphomimetic substitution of Ser_PKA_ with an acidic residue locks Drp1 into a partially inhibited state, arresting the enzyme at a different stage of its subcellular translocation cycle.

Enhanced colocalization or cofractionation of Drp1 with mitochondria has previously been interpreted as evidence for Drp1 activation (e.g. [17],[20],[46]). Our data and those of Zunino et al. [44] argue that Drp1 regulation is more complex, in that mitochondria-associated pools of Drp1 may sometimes be inactive. Similar considerations apply to higher order oligomeric assembly of Drp1, which is clearly required for its function as a mechanoenzyme [28]. The crosslinking and particle tracking data presented here indicate that excessive oligomerization of Drp1 into particles unable to constrict and sever mitochondria occurs as a consequence of Ser_PKA_ phosphorylation or mutation of the GTPase domain.

In support of a model in which PKA/AKAP1 fuses mitochondria by accumulating Drp1 in inactive superstructures (Figure 10C), recent studies on the mechanism of action of dynamin suggest that the endocytosis motor assembles into relatively short oligomers (3 to 4 rungs of a spiral) before GTP hydrolysis-driven disassembly leads to membrane destabilization and scission. In contrast, disassembly of larger dynamin oligomers (assembled in the absence of GTP) does not effectively mediate membrane scission [47],[48].

Outer mitochondrial PKA-induced super-oligomerization of Drp1 could inhibit cell death by several, non-mutually exclusive mechanisms. For instance, mitochondrial networks resulting from unopposed fusion can sustain higher metabolic activity [49], are relatively resistant to Bax insertion and cytochrome C release [50], and may also be more effective at sequestering cytotoxic calcium and reactive oxygen species [51],[52]. More directly, PKA-mediated depletion of the cytosolic Drp1 pool could potentially interfere with pathological Drp1 activation by sumoylation [43] and nitrosylation [15] and compete with Drp1 recruitment into Bax/Bak positive foci during apoptosis [53]. The interplay between multi-site phosphorylation and other posttranslational modifications of Drp1 in the regulation of mitochondrial homeostasis and cell death is undoubtedly complex and will require further attention.

## Materials and Methods

### Antibodies

The following antibodies were used: rabbit anti-GFP (ab290, Abcam), mouse IgG_1_ anti-MTCO2 (cytochrome oxidase subunit II, Neomarkers), rabbit anti-ERK (Santa Cruz), mouse anti-phospho-Ser_PKA_ Drp1 [22], rabbit anti-phospho-Ser_CDK_ Drp1 (Ser616, Cell Signaling), rabbit anti-LacZ and mouse IgG_2a_ anti-V5 epitope tag (Invitrogen), mouse anti-neurofilament (2H3, Developmental Studies Hybridoma Bank, Iowa City), and mouse anti-MAP2B (BD Transduction Laboratories). For immunofluorescence staining, Alexa fluorophore-coupled secondary antibodies were purchased from Invitrogen. Infrared fluorophore-coupled secondary antibodies for quantitative immunoblot analysis were purchased from LI-COR Biosciences (Lincoln, NE).

### cDNA and shRNA Vectors

The core domain of rat AKAP1 that is present in all published splice variants (residues 1–524) was isolated by reverse transcriptase PCR and fused to the N terminus of EGFP. The PKA-binding deficient mutant I310P, L316P was generated by mutagenesis according to the QuikChange protocol (Stratagene). N-terminally GFP tagged PKA, PKI [54], and PfARP_32–239_ [55] were modified by the addition of N-terminal OMM targeting sequences: hexokinase I_1–30_ for PKA and MAS70p_1–29_ for PKI. For the V5-tagged AKAP1 constructs, the AKAP1 cDNA was excised from the GFP constructs with BglII and SalI and ligated into the pcDNA3.1HisV5 vector digested with BamHI and XhoI. AKAP1 and Fis1 were silenced by H1-promoter-driven expression of shRNAs [56] (pSUPER plasmid [57] or lentivirus [58]). 19 b target sites in the mRNAs were (numbering relative to translation start site in rat mRNAs): AKAP1/#1: 780–788, AKAP1/#4: 740–758, Fis1/#3: 315–333, Fis1/#4: 421–439. The nonspecific control shRNA had a similar base composition but was randomized for no more than 14 consecutive matches to any mammalian mRNA. The Fis1 and control shRNAs were described previously [21]. Drp1 expression plasmids encoded rat splice variant 1 with an N-terminal EGFP tag under CMV promoter control, as well as Drp1-directed shRNA driven by the H1 promoter [18]. GFP-Drp1 was rendered RNAi resistant and coding mutations were incorporated by site-directed mutagenesis [18]. All Drp1 transfection experiments in this article involved concomitant silencing of the endogenous protein.

### Western Blot and Immunoprecipitation

For analysis of Drp1 phosphorylation, COS cells were cotransfected with GFP-tagged AKAP1 and Drp1 at 2∶3 plasmid mass ratios using Lipofectamine 2000. After 24 h, cells were stimulated with various concentrations of forskolin/rolipram for 45 min, lysed in SDS sample buffer containing 2 mM EDTA and 1 µM microcystin, and sonicated with a probe tip to shear DNA. For AKAP1 immunoprecipitation, PC12 cells were transfected with AKAP1 WT- or AKAP1ΔPKA-GFP. After 48 h, AKAP1 was immunoprecipitated with GFP antibodies and protein A-agarose beads essentially as described [59]. Protein samples were resolved on 8% or 10% polyacrylamide gels and transferred to nitrocellulose membrane, followed by antibody detection using a LI-COR Odyssey infrared fluorescence scanner. Band intensities were quantified using the ImageJ gel analysis macro set, normalizing to loading controls in the same lane.

### Intact Cell Crosslinking

COS cells were transfected with triple HA-tagged Drp1 and either omGFP or omPKA expressing plasmids at 3∶1 mass ratio and cultured for 24 h. After a wash with PBS, cells were incubated with HBSS containing up to 0.5 mM dithiobismaleimidoethane (DTME, from freshly prepared 50× stocks in DMSO) for 5 min at 37°C. The HBSS/DTME was removed and cells were lysed in buffer containing 60 mM Tris pH 6.8 and 2% SDS. Insoluble debris was removed by centrifugation (10 min at 14,000 x g) and cleared lysates (300 µl) were layered onto 1 ml of a 300 mM sucrose cushion and subjected to ultracentrifugation (30 min at 300,000 x g). Pellets were dissolved in SDS sample buffer containing 5% β-mercaptoethanol to cleave the crosslinker and analyzed by SDS-PAGE alongside an aliquot of the lysate prior to ultracentrifugation.

### Neuronal Survival Assays

Hippocampal neurons from E18 rat embryos [60] were cultured in Neurobasal medium with B27 supplement (Gibco) and transduced with lentivirus or transfected using LipofectAmine 2000 (0.15%, 2 µg/ml DNA) at 10–21 d in vitro. For survival assays based on counting transfected neurons or apoptotic nuclei, GFP- or β-galactosidase-expressing plasmids were cotransfected. After 3 to 5 d, cultures were challenged with rotenone (400 nM continuous) and fixed with 3.7% paraformaldehyde and processed for immunofluorescence staining for the transfection marker (αGFP and αLacZ) and neurofilament protein as a neuronal marker (2H3). To score survival, transfection marker-positive neurons with intact processes were counted in quadruplicate wells of a 24-well plate. Apoptosis was quantified as the percentage of transfected neurons with condensed, irregular, or fragmented nuclei (labeled with 1 µg/ml Hoechst 33342). All survival assays based on counting neurons were performed blind to the experimental conditions.

### Mitochondrial Morphology Assays in Cell Culture

HeLa cells, PC12 cells, and hippocampal neurons cultured on, respectively, untreated, collagen-, and poly-D-lysine-coated, chambered No. 1 cover glasses (20 mm^2^ chamber, Nalge Nunc) were infected with lentivirus or transfected using LipofectAmine 2000 as above. Hippocampal cultures were infected or transfected at 8 to 10 d in vitro and analyzed 3 to 5 d later. For live cell imaging 24 to 96 h post-transfection, cells were incubated with 100 nM TMRM for 30 min at 37°C to visualize mitochondria, and images through the midplane of the soma were captured using a Zeiss LSM 510 laser-scanning confocal microscope. For analysis of fixed cells, cultures were subjected to immunofluorescence staining with antibodies to cytochrome oxidase II (HeLa cells only) and GFP [22], and images were captured with a Leica epifluorescence microscope.

Mitochondrial morphology was scored by reference image-based and software-based methods. For the former, coded images were assigned scores from 0 to 4 by comparison to a set of reference images with increasing degrees of mitochondrial elongation and clustering (Figure S1A). For automated morphometry, images were processed using ImageJ software and plugins, involving either “rolling ball” background subtraction or deblurring by 2-D deconvolution with a computed point spread function. Using a custom-written ImageJ macro, processed images were converted to binary (black and white) images by auto-thresholding, and mitochondrial particles were analyzed for length, width, area (a), and perimeter (p) [22]. Metrics that reliably reported the effects of manipulating components of the mitochondrial fission/fusion machinery (e.g. wild-type or dominant-negative Drp1 expression, Fis1 or Drp1 RNAi) included form factor (p^2^/(4π*a)) and cumulative area:perimeter ratio (Σa/Σp). The form factor is reported as an average of all particles in a region-of-interest (ROI), has a minimum value of 1 (for perfect circles), and captures well the transition from punctiform to elongated, complex shaped, but still isolated mitochondria. The cumulative area:perimeter ratio is computed as the summed particle area in a ROI divided by the summed particle perimeter (including the perimeter of enclosed spaces or “holes”). This metric is particularly effective at detecting the transition from elongated, isolated mitochondria to a reticular network of interconnected mitochondria. Colocalization of GFP-Drp1 with mitochondria (Figure 9C) was quantified as the Manders' coefficient using the JaCoP plug-in for ImageJ [61].

### In Vivo Mitochondrial Morphology Analysis

All animal procedures were approved and carried out according to the Institutional Animal Care and Use Committee (IACUC) at the University of Iowa. Adult male Lewis rats were anesthetized with 91/9.1 mg/kg of ketamine/xylazine and placed in a stereotaxic apparatus. Concentrated stocks (∼10^8^ particles/ml) of lentivirus (feline immunodeficiency virus), prepared by the University of Iowa Viral Vector Core, were bilaterally injected into the striatum and hippocampus using a 10 µl Hamilton syringe with a 30° beveled 30 gauge needle. Virus expressing mitochondria-targeted GFP (via residues 1–31 of cytochrome oxidase subunit VIII) and AKAP1-GFP were delivered into the left and right hemisphere, respectively. The following coordinates were used with the incisor bar at 3.3 (from bregma): Striatum, AP = 0, ML = ±3.5, DV = −4.5; Hippocampus, AP = −4.0, ML = ±2.0, DV = −3.5. Animals were sacrificed 7–14 d later and transcardially perfused with 4% paraformaldehyde. 40 µm thick coronal cryostat sections were processed for indirect immunofluorescence with antibodies to GFP and MAP2B and counterstained for nuclei with TOPRO-3. Ten to 22 confocal z-sections of infected neurons, 0.8 µm apart, were captured and analyzed for mitochondrial shape by digital morphometry as described above.

### Fluorescence Recovery after Photobleaching

Hela and PC12 cells were grown on collagen-coated, chambered No. 1 cover glasses (Nunc, Thermo Fisher), transfected 24 to 48 h, and stained with MitoTracker Deep Red 30 min prior to analysis. Cells with non-mitochondrial GFP-Drp1 aggregates, a sign of overexpression, were excluded from the analysis. Using the 488 nm laser line of a Zeiss LSM 510 confocal microscope, an approx. 5×5 µm region of mitochondria-associated GFP-Drp1 was bleached and fluorescence recovery was tracked by capturing images every 5 s for 5 min. Image stacks were analyzed, and recovery curves normalized to pre-bleach intensity and corrected for acquisition bleach [27] were approximated by single and double exponential recovery equations using a custom-written ImageJ macro, yielding 50% recovery time constant (t_1/2_) and mobile fraction (mFx). Drp1 dynamics followed double exponential recovery kinetics with *R* ^2^ values of generally greater than 0.99. Control experiments, in which bleach recovery of cytosolic and mitochondrial Drp1 was tracked separately by masking the GFP channel with the MitoTracker channel, showed that mitochondrial masking does not significantly affect the results (unpublished data).

### GFP-Drp1 Particle Tracking

HeLa cells cotransfected with GFP-Drp1, dsRed2/mito, and either empty vector or PKA catalytic subunit at 15∶4∶1 plasmid ratios were subjected to time lapse imaging using the 63× objective of a motorized Leica AF6000 epifluorescence microscope under temperature (37°C) and CO_2_ (5%) control. Frames were captured at 30 s intervals for 60 to 90 min, contrast-enhanced (contrast-limited adaptive histogram equalization [CLAHE] plugin for ImageJ), and analyzed with the Particle Tracker plugin for ImageJ [31]. Text files containing particle trajectories were parsed with a custom-written macro to obtain mean particle lifetimes and lifetime histograms.

### Subcellular Fractionation

COS cells expressing GFP-Drp1 with or without omPKA were permeabilized in 0.5 mg/ml digitonin, 100 mM KCl, 20 mM HEPES pH 7.4, 1 mM EDTA, 1 mM EGTA, 1 mM benzamidine, 5 µg/ml leupeptin, 1 mM dithiotreitol (DTT), and 1 mM phenylmethylsulfonyl fluoride (PMSF). Cytosolic and heavy membrane fractions were prepared by centrifugation (10 min, 20,000 x g, 4°C).

### Statistical Analysis

Data were analyzed by Student's *t* test (two-tailed) for single comparisons and by one-way analysis of variance (ANOVA) followed by pairwise Bonferroni post hoc tests for multiple comparisons.

## Supporting Information

Figure S1 Reference image-based and automated methods yield similar mitochondrial morphology scores. (A) Confocal images of TMRM-stained mitochondria in hippocampal neurons were used as reference images for quantification of mitochondrial morphology (increasing length scores from 0 to 4). (B, C) Comparison of blinded reference image-based length scores (B) and digital morphometry (C) applied to confocal images of hippocampal neurons expressing Bβ2- or AKAP1-directed or nonsense (NS) shRNAs (means ± s.d. of 12–28 neurons). The cumulative area:perimeter ratio (Σa/Σp) can account for mitochondrial clusters and achieves significance values approaching that of the reference-image-based method. (D) Cell-by-cell correlation of reference-image-based length scores and cumulative area:perimeter ratios in neurons expressing Bβ1 or Bβ2 and Bcl2. Small symbols represent values from individual neurons; the large symbols are population means (± s.d.).
(0.38 MB TIF)

Figure S2 A point mutation and two shRNAs interfere with AKAP1 function/expression and Drp1 shRNA permits assembly of pure GFP-Drp1 oligomers. (A) Wild-type (WT) and mutant (ΔPKA  =  I310P,L316P) AKAP1-GFP were immunoprecipitated via GFP from transfected COS cells and analyzed for association with the PKA regulatory subunit RIIα; the asterisk indicates immunoglobulin heavy chain. (B) Hippocampal cultures were cotransfected with a 1∶1∶2 mass ratio of plasmids expressing the indicated target proteins fused to the N terminus of firefly luciferase (luc), *Renilla* luciferase, and shRNAs (NS, nonsense). Knockdown was assessed 3 d later by dual-luciferase assays. AKAP1-directed shRNAs were as effective as a positive control shRNA targeting the luc coding sequence. (C) PC12 cells transfected with the indicated shRNA plasmids were probed for endogenous AKAP1 and ERK1/2 expression (duplicate cultures). At least two AKAP1 splice variants are expressed in PC12 cells, with the ∼120 kD variant predominating. (D) HeLa cells were transfected with GFP-Drp1 expression plasmids that include (+) or exclude (−) a H1 promoter-shRNA cassette to silence endogenous Drp1, and GFP immunoprecipitates were probed with Drp1 antibodies. Endogenous Drp1 assembles with (RNAi-resistant) GFP-Drp1 only in the absence of Drp1-directed shRNA.
(0.25 MB TIF)

Figure S3 AKAP1 regulates mitochondrial shape in dendrites of hippocampal neurons. Hippocampal cultures were transfected with mitochondrial GFP and the indicated constructs at 1∶4 mass ratio at 10 DIV and fixed 3 d later for analysis of mitochondrial shape in dendrites. (A) Representative epifluorescence images and graphs (B) showing mitochondrial form factor (mean ± s.e.m. of 40–50 neurons from a representative experiment). *p* values refer to comparisons to control (nonsense shRNA) transfected conditions.
(0.26 MB TIF)

Figure S4 Rescue of rat AKAP1 knockdown-induced mitochondrial fragmentation by expression of human AKAP1. PC12 cells were cotransfected with mitochondrial GFP, the indicated shRNAs, and either human AKAP1 cDNA or empty vector, fixed after 3 d, and analyzed by epifluorescence microscopy. (A) Representative images (green, mitochondria; blue, DNA) and graphs (B) depicting mitochondrial shape analysis from a representative experiment (mean ± s.e.m. of 300–400 cells per condition).
(1.31 MB TIF)

Figure S5 Neuronal death due to AKAP1 silencing cannot be rescued by inhibiting mitochondrial fission. Hippocampal cultures were cotransfected with plasmids expressing AKAP1-directed or control shRNAs and the indicated rescue plasmids and analyzed after 5 d for mitochondrial morphology (A) and apoptosis (B) (means ± s.e.m. of 3–5 experiments). Bcl2, but neither dominant-negative Drp1 nor Fis1 RNAi, attenuates apoptosis associated with AKAP1 knockdown.
(0.14 MB TIF)

Figure S6 Protonophore-mediated mitochondrial fragmentation does not affect Drp1 dynamics. HeLa cells expressing GFP-Drp1 were treated with either vehicle (basal) or 10 µM carbonylcyanide p-trifluoromethoxyphenylhydrazone (FCCP) and 2 µM oligomycin (prevents ATP depletion due to reversal of the F1 ATP synthase) for 1 to 4 h, during which time Drp1 turnover was measured by FRAP. FCCP leads to dramatic mitochondrial fragmentation as quantified by form factor (A) but has no effect on Drp1 recovery (B, representative frames; C, average recovery curves [± s.e.m.] from a representative experiment).
(1.53 MB TIF)

Figure S7 AKAP1 enhances forskolin-stimulated Drp1 phosphorylation in PC12 cells. PC12 cells cotransfected with GFP-Drp1 and either empty vector, AKAP1, or AKAP1 ΔPKA were stimulated for 45 min with the indicated concentrations of forskolin in the presence of rolipram (2 µM). Total cell lysates were probed for phospho-Ser_PKA_ Drp1 (pDrp1) and total Drp1 on the same blot using a dual-channel infrared imager. Drp1 phosphorylation was quantified as the ratio of phospho- to total Drp1 signals normalized to the highest value. Shown is one experiment representative of two.
(0.11 MB TIF)

Figure S8 Elongation of dendritic mitochondria by AKAP1 requires Drp1 Ser_PKA_. Primary hippocampal neurons cotransfected with wild-type or Ser_PKA_-mutant GFP-Drp1 and either vector, wild-type, or PKA binding-deficient (ΔPKA) AKAP1 were analyzed for morphology of dendritic mitochondria after immunofluorescence staining for the OMM-protein TOM20 (representative images in A). (B) and (C) show mitochondrial form factor and length, respectively, from the same set of neurons (means ± s.e.m. of 12–24 neurons per condition from two culture dates).
(0.36 MB TIF)

Video S1 AKAP1 slows Drp1 fluorescence recovery. Coexpression with AKAP1 slows recovery of GFP-Drp1 in the photobleached square compared to when either protein is mutated.
(0.80 MB MPG)

Video S2 A mutation in the GTPase domain slows Drp1 fluorescence recovery. FRAP kinetics of the T55D mutant are attenuated compared to wild-type GFP-Drp1.
(0.89 MB MPG)

Video S3 PKA and GTPase mutation extend the lifetime of mitochondrial Drp1 punctae. Coexpression with the PKA catalytic subunit or T55D substitution leads to the accumulation of large GFP-Drp1 punctae that turn over more slowly than wild-type Drp1 without PKA.
(1.87 MB MPG)

## Abbreviations

AKAP1: A kinase anchoring protein 1
CAMKI: Ca^2+^/calmodulin-dependent protein kinase I
Drp1: dynamin-related protein 1
DTME: dithiobismaleimidoethane
FRAP: fluorescence recovery after photobleaching
GED: GTPase effector domain
OMM: outer-mitochondrial membrane
omPKA: outer mitochondrial PKA
omPKI: outer mitochondrial protein kinase A inhibitor
OPA1: optic atrophy 1
PKA: protein kinase A
PP: protein phosphatase
TMRM: tetramethylrhodamine methyl ester
