# Supplementary figures and images for: Mechanism of Neuroprotective Mitochondrial Remodeling by PKA/AKAP1
Source: PLoS Biol. 2011 Apr 19;9(4):e1000612. doi: 10.1371/journal.pbio.1000612 (PMC3079583; doi:10.1371/journal.pbio.1000612)

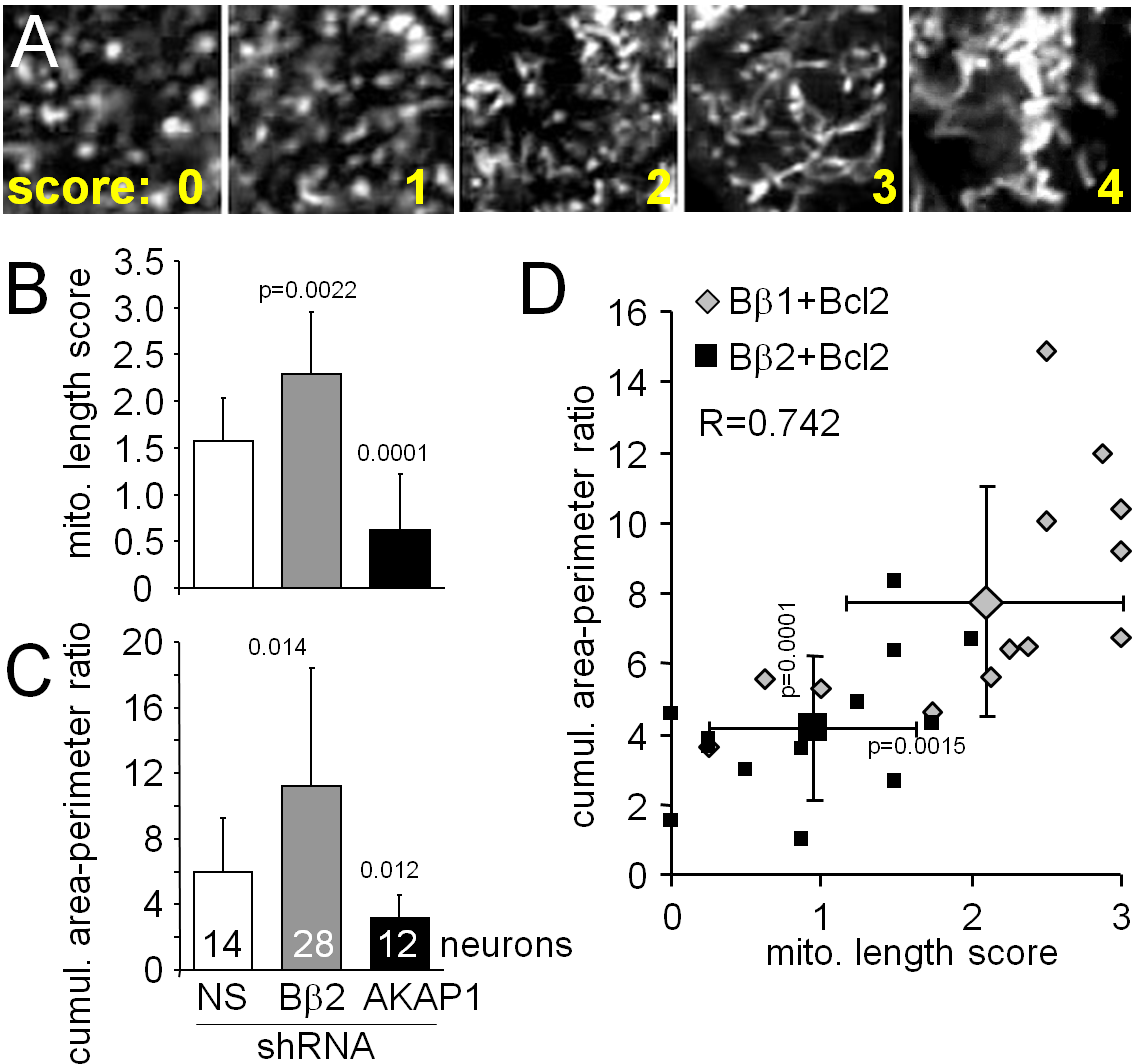

Supplement: Figure S1 — Reference image-based and automated methods yield similar mitochondrial morphology scores. (A) Confocal images of TMRM-stained mitochondria in hippocampal neurons were used as reference images for quantification of mitochondrial morphology (increasing length scores from 0 to 4). (B, C) Comparison of blinded reference image-based length scores (B) and digital morphometry (C) applied to confocal images of hippocampal neurons expressing Bβ2- or AKAP1-directed or nonsense (NS) shRNAs (means ± s.d. of 12–28 neurons). The cumulative area:perimeter ratio (Σa/Σp) can account for mitochondrial clusters and achieves significance values approaching that of the reference-image-based method. (D) Cell-by-cell correlation of reference-image-based length scores and cumulative area:perimeter ratios in neurons expressing Bβ1 or Bβ2 and Bcl2. Small symbols represent values from individual neurons; the large symbols are population means (± s.d.). (0.38 MB TIF) [file pbio.1000612.s001.tif]

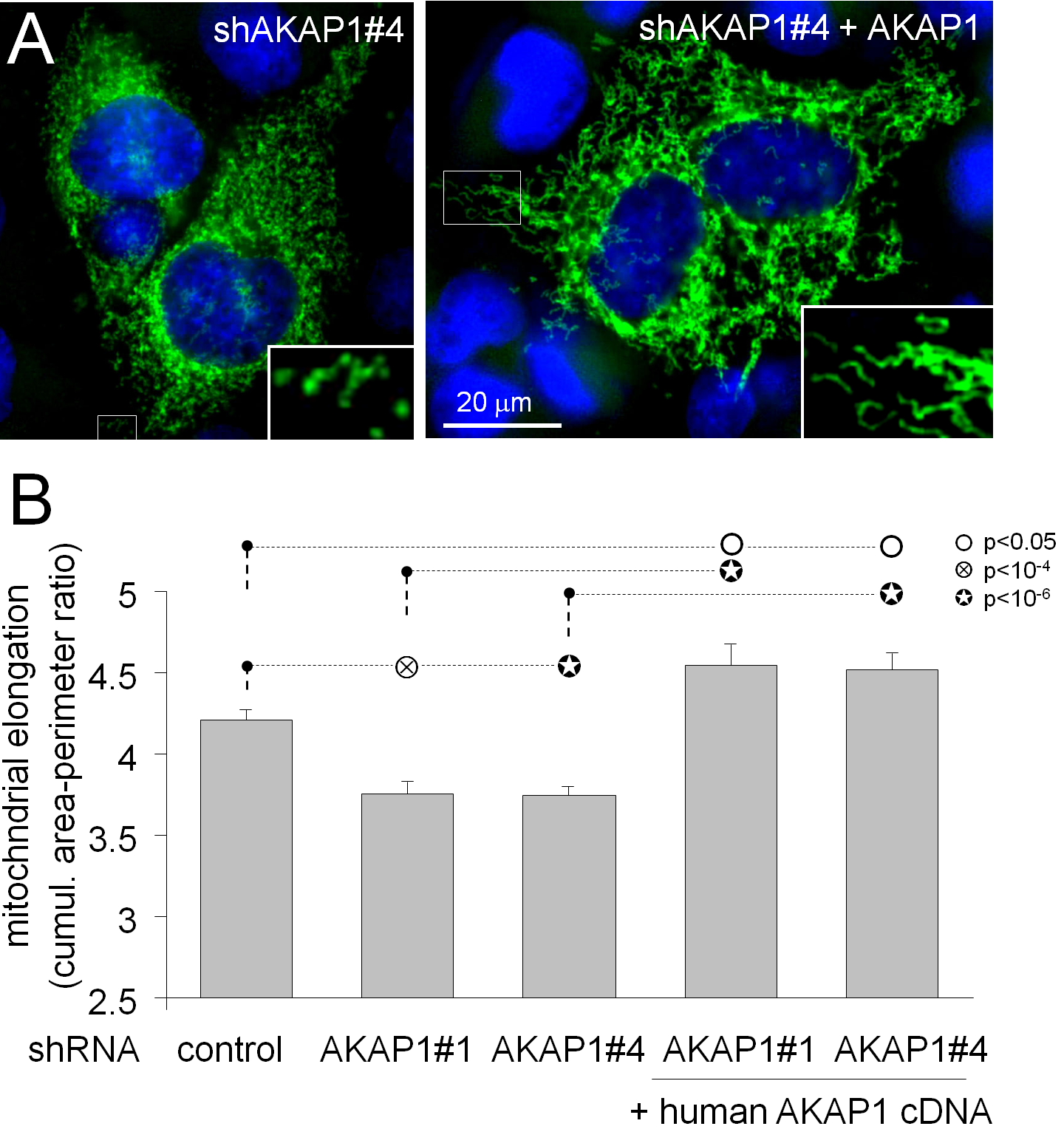

Supplement: Figure S4 — Rescue of rat AKAP1 knockdown-induced mitochondrial fragmentation by expression of human AKAP1. PC12 cells were cotransfected with mitochondrial GFP, the indicated shRNAs, and either human AKAP1 cDNA or empty vector, fixed after 3 d, and analyzed by epifluorescence microscopy. (A) Representative images (green, mitochondria; blue, DNA) and graphs (B) depicting mitochondrial shape analysis from a representative experiment (mean ± s.e.m. of 300–400 cells per condition). (1.31 MB TIF) [file pbio.1000612.s004.tif]

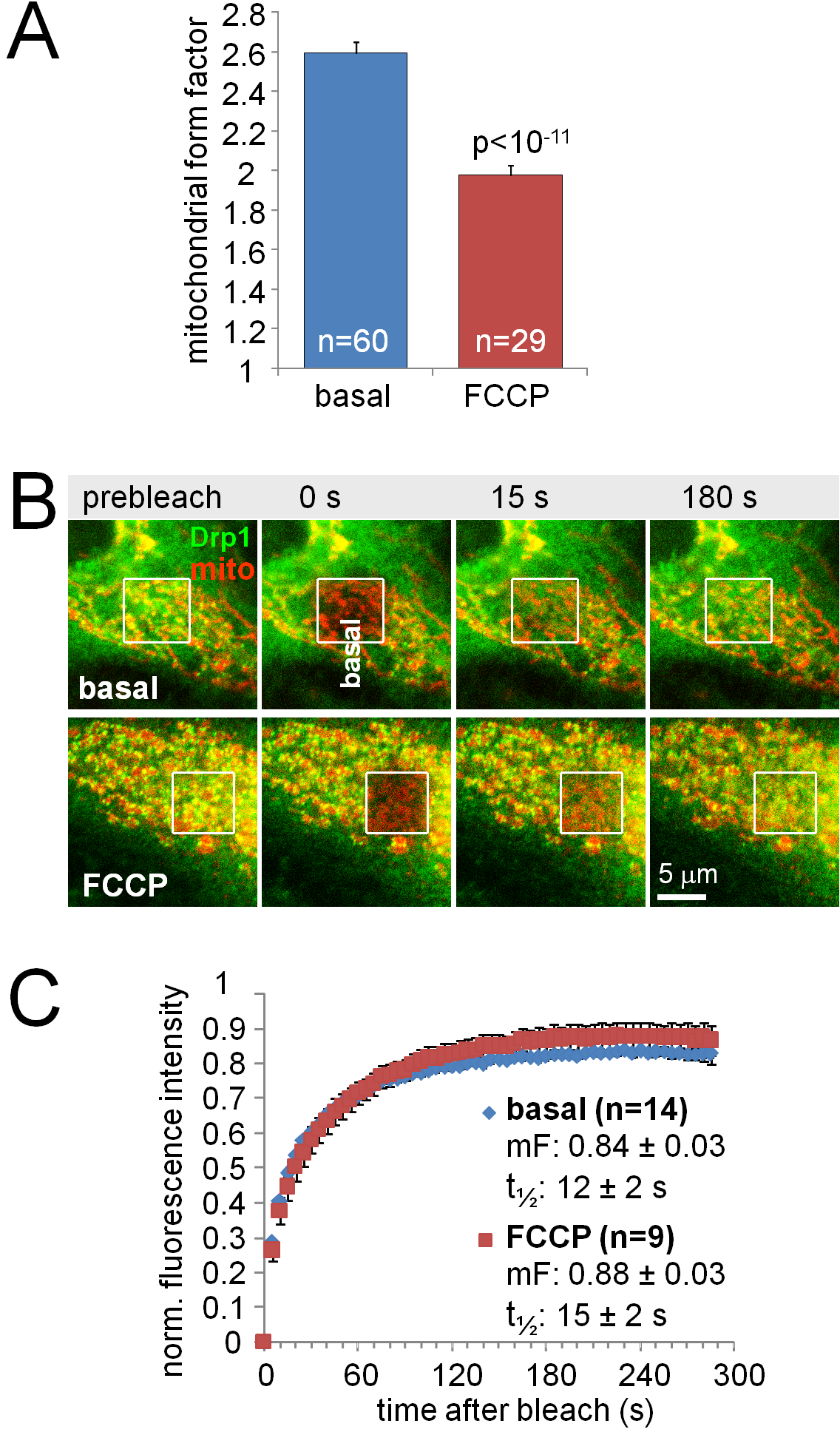

Supplement: Figure S6 — Protonophore-mediated mitochondrial fragmentation does not affect Drp1 dynamics. HeLa cells expressing GFP-Drp1 were treated with either vehicle (basal) or 10 µM carbonylcyanide p-trifluoromethoxyphenylhydrazone (FCCP) and 2 µM oligomycin (prevents ATP depletion due to reversal of the F1 ATP synthase) for 1 to 4 h, during which time Drp1 turnover was measured by FRAP. FCCP leads to dramatic mitochondrial fragmentation as quantified by form factor (A) but has no effect on Drp1 recovery (B, representative frames; C, average recovery curves [± s.e.m.] from a representative experiment). (1.53 MB TIF) [file pbio.1000612.s006.tif]

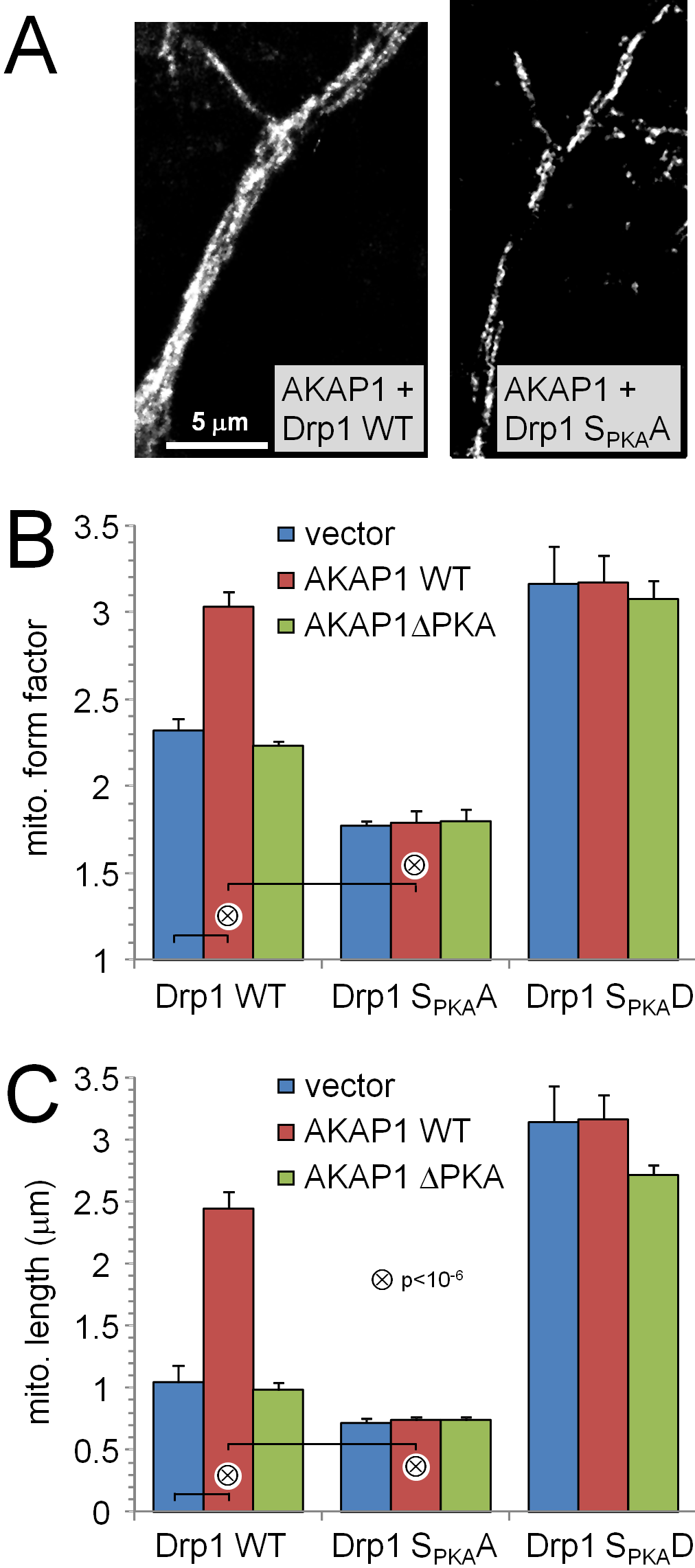

Supplement: Figure S8 — Elongation of dendritic mitochondria by AKAP1 requires Drp1 SerPKA. Primary hippocampal neurons cotransfected with wild-type or SerPKA-mutant GFP-Drp1 and either vector, wild-type, or PKA binding-deficient (ΔPKA) AKAP1 were analyzed for morphology of dendritic mitochondria after immunofluorescence staining for the OMM-protein TOM20 (representative images in A). (B) and (C) show mitochondrial form factor and length, respectively, from the same set of neurons (means ± s.e.m. of 12–24 neurons per condition from two culture dates). (0.36 MB TIF) [file pbio.1000612.s008.tif]
